# A metabolic constraint in *de novo* NAD^+^ synthesis drives mucosal inflammation in IBD

**DOI:** 10.1093/ecco-jcc/jjag043

**Published:** 2026-06-01

**Authors:** Lina Wehkamp, Danielle M M Harris, Na-mi Kim, Abrar I Alsaadi, Qicong Wu, Mhmd Oumari, Jan Taubenheim, Valery Volk, Graziella Credidio, Eric Koncina, Pranab K Mukherjee, Florian Tran, Taous Mekdoud, Meiping Yu, Laura K Sievers, Polychronis Pavlidis, Nick Powell, Shihan Wang, Ivan Fung, Georg H Waetzig, Christel Rousseaux, Pierre Desreumaux, Florian Rieder, Elisabeth Letellier, Silvio Waschina, Thomas F Meyer, Timon Adolph, Geert D’Haens, Christoph Kaleta, Friedrich Feuerhake, Bram Verstockt, Melanie R McReynolds, Philip Rosenstiel, Stefan Schreiber, Konrad Aden

**Affiliations:** Institute of Clinical Molecular Biology, Christian-Albrechts-University and University Hospital Schleswig-Holstein, Kiel, 24105, Germany; Department of Internal Medicine I, Christian-Albrechts-University and University Hospital Schleswig-Holstein, Kiel, 24105, Germany; Institute of Clinical Molecular Biology, Christian-Albrechts-University and University Hospital Schleswig-Holstein, Kiel, 24105, Germany; Department of Internal Medicine I, Christian-Albrechts-University and University Hospital Schleswig-Holstein, Kiel, 24105, Germany; Institute of Clinical Molecular Biology, Christian-Albrechts-University and University Hospital Schleswig-Holstein, Kiel, 24105, Germany; Department of Internal Medicine I, Christian-Albrechts-University and University Hospital Schleswig-Holstein, Kiel, 24105, Germany; Department of Biochemistry and Molecular Biology, The Huck Institute of the Life Sciences, Pennsylvania State University, University Park, PA, 16802, United States; Institute of Clinical Molecular Biology, Christian-Albrechts-University and University Hospital Schleswig-Holstein, Kiel, 24105, Germany; Department of Internal Medicine I, Christian-Albrechts-University and University Hospital Schleswig-Holstein, Kiel, 24105, Germany; Institute of Clinical Molecular Biology, Christian-Albrechts-University and University Hospital Schleswig-Holstein, Kiel, 24105, Germany; Department of Internal Medicine I, Christian-Albrechts-University and University Hospital Schleswig-Holstein, Kiel, 24105, Germany; Institute of Experimental Medicine, Christian-Albrechts-University and University Hospital Schleswig-Holstein, Kiel, 24105, Germany; Institute for Pathology, Hannover Medical School, Hannover, 30625, Germany; Institute of Clinical Molecular Biology, Christian-Albrechts-University and University Hospital Schleswig-Holstein, Kiel, 24105, Germany; Department of Life Sciences and Medicine, University of Luxembourg, L-4365 Belvaux, Luxembourg; Department of Inflammation and Immunity, Lerner Research Institute, Cleveland Clinic, Cleveland, OH, 44195, United States; Centre for Inflammation Biology and Cancer Immunology, King’s College London, London, SE1 1UL, United Kingdom; Institute of Clinical Molecular Biology, Christian-Albrechts-University and University Hospital Schleswig-Holstein, Kiel, 24105, Germany; Department of Internal Medicine I, Christian-Albrechts-University and University Hospital Schleswig-Holstein, Kiel, 24105, Germany; Institute of Clinical Molecular Biology, Christian-Albrechts-University and University Hospital Schleswig-Holstein, Kiel, 24105, Germany; Department of Internal Medicine I, Christian-Albrechts-University and University Hospital Schleswig-Holstein, Kiel, 24105, Germany; Institute of Clinical Molecular Biology, Christian-Albrechts-University and University Hospital Schleswig-Holstein, Kiel, 24105, Germany; Department of Internal Medicine I, Christian-Albrechts-University and University Hospital Schleswig-Holstein, Kiel, 24105, Germany; Institute of Clinical Molecular Biology, Christian-Albrechts-University and University Hospital Schleswig-Holstein, Kiel, 24105, Germany; Department of Internal Medicine I, Christian-Albrechts-University and University Hospital Schleswig-Holstein, Kiel, 24105, Germany; Center for Global Translational Inflammatory Bowel Disease Research, Cleveland Clinic, Cleveland, OH 44195, United States; School of Immunology and Microbial Sciences, King’s College London, London, SE1 9RT, United Kingdom; Division of Digestive Diseases, Faculty of Medicine, Imperial College London, London, W2 1NY, United Kingdom; Institute of Clinical Molecular Biology, Christian-Albrechts-University and University Hospital Schleswig-Holstein, Kiel, 24105, Germany; Tytgat Institute for Liver and Intestinal Research, Amsterdam University Medical Centers, Amsterdam, 1105 BK, The Netherlands; Institute of Clinical Molecular Biology, Christian-Albrechts-University and University Hospital Schleswig-Holstein, Kiel, 24105, Germany; CONARIS Research Institute AG, Kiel, 24118, Germany; Intestinal Biotech Development, Lille, 59000, France; Intestinal Biotech Development, Lille, 59000, France; Department of Inflammation and Immunity, Lerner Research Institute, Cleveland Clinic, Cleveland, OH, 44195, United States; Centre for Inflammation Biology and Cancer Immunology, King’s College London, London, SE1 1UL, United Kingdom; Department of Gastroenterology, Hepatology and Nutrition, Digestive Disease Institute, Cleveland Clinic, Cleveland, OH 44195, United States; Department of Life Sciences and Medicine, University of Luxembourg, L-4365 Belvaux, Luxembourg; Institute for Human Nutrition and Food Science, Nutriinformatics, Kiel University, Kiel, 24105, Germany; Institute of Clinical Molecular Biology, Christian-Albrechts-University and University Hospital Schleswig-Holstein, Kiel, 24105, Germany; Department of Molecular Biology, Max Planck Institute for Infection Biology, Berlin, 10117, Germany; Department of Medicine I, Gastroenterology, Hepatology & Metabolism, Medical University Innsbruck, Innsbruck, 6020, Austria; Department of Gastroenterology and Hepatology, Amsterdam University Medical Centers, Amsterdam, 1081HZ, The Netherlands; Institute of Experimental Medicine, Christian-Albrechts-University and University Hospital Schleswig-Holstein, Kiel, 24105, Germany; Institute for Pathology, Hannover Medical School, Hannover, 30625, Germany; Department of Gastroenterology and Hepatology, University Hospitals Leuven, Katholieke Universiteit Leuven, Leuven, 3000, Belgium; Department of Chronic Diseases and Metabolism, Katholieke Universiteit Leuven, Leuven, 3000, Belgium; Department of Biochemistry and Molecular Biology, The Huck Institute of the Life Sciences, Pennsylvania State University, University Park, PA, 16802, United States; Institute of Clinical Molecular Biology, Christian-Albrechts-University and University Hospital Schleswig-Holstein, Kiel, 24105, Germany; Department of Internal Medicine I, Christian-Albrechts-University and University Hospital Schleswig-Holstein, Kiel, 24105, Germany; Institute of Clinical Molecular Biology, Christian-Albrechts-University and University Hospital Schleswig-Holstein, Kiel, 24105, Germany; Department of Internal Medicine I, Christian-Albrechts-University and University Hospital Schleswig-Holstein, Kiel, 24105, Germany; Institute of Clinical Molecular Biology, Christian-Albrechts-University and University Hospital Schleswig-Holstein, Kiel, 24105, Germany; Department of Internal Medicine I, Christian-Albrechts-University and University Hospital Schleswig-Holstein, Kiel, 24105, Germany

**Keywords:** IBD, tryptophan, quinolinic acid, NAD, metabolism

## Abstract

**Background:**

Inflammatory bowel disease (IBD) is associated with energy deficiency and perturbed metabolism of the essential amino acid tryptophan (Trp).

**Objective:**

We aimed to determine whether excessive Trp degradation fuels or compensates for inflammation through *de novo* nicotinamide adenine dinucleotide (NAD^+^) synthesis.

**Design:**

A prospective systems medicine approach (metabolomics, transcriptomics) was employed longitudinally in patients with advanced IBD therapy. Findings were validated with targeted Trp metabolomics in experimental colitis and *in vitro* in fibroblasts, intestinal epithelial cells (IECs), and peripheral blood mononuclear cells (PBMCs).

**Results:**

Active IBD is marked by enhanced Trp degradation driven by inflammatory cytokines through the JAK/STAT pathway. Trp catabolism results in accumulation of quinolinic acid (QA) and NAD^ + ^depletion due to reduced expression of QPRT, the enzyme converting QA to NAD^+^. QPRT knockdown enhances inflammation, while NAD^ + ^precursor supplementation (e.g. nicotinamide riboside [NR]) restores cellular energy and reduces inflammation *in vitro* and in dextran sodium sulfate (DSS)-induced colitis.

**Conclusion:**

A metabolic bottleneck at QPRT prevents efficient NAD^+^ synthesis from Trp in IBD, sustaining inflammation. Restoring NAD^ + ^is a promising therapeutic strategy.

## Graphical Abstract

**Graphical Abstract:**
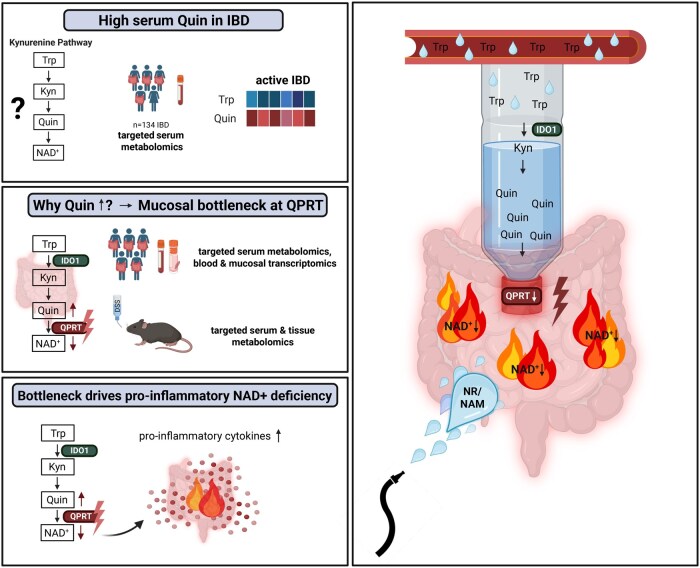
Created with Biorender.com.

## 1. Introduction

Current inflammatory bowel disease (IBD) therapies target immune pathways such as TNFα, IL-23, JAK/STAT, and IL-6, yet up to 40% of patients fail to respond, underscoring the need for a deeper pathophysiological understanding and new therapeutic strategies.^1^^,^^2^ Disturbed metabolism of the essential amino acid tryptophan (Trp) has emerged as an overarching hallmark of chronic inflammatory diseases.^3^ Trp is primarily metabolized via the kynurenine pathway (KP), a pathway strongly activated during inflammation, with >90% of Trp catabolized by the initial enzyme indoleamine 2,3-dioxygenase 1 (IDO1).^4^ This results in production of various bioactive metabolites, including nicotinamide adenine dinucleotide (NAD^+^). Enhanced KP activity has been observed in IBD, correlating with endoscopic and clinical disease activity.^3^^,^^5–7^ Quinolinic acid (QA), a key intermediate in the NAD^++^ biosynthetic branch of the KP, is elevated in serum during IBD flares, though the mechanisms underlying its accumulation remain unclear.^7^^,^^8^

Despite increased Trp breakdown, NAD^+^ depletion persists in the inflamed mucosa. This is clinically relevant, as energy deficiency is a known driver of epithelial dysfunction and immune activation in IBD.^9^ NAD^+^ is an essential cofactor in energy metabolism, mitochondrial function, and redox balance and plays a critical role in maintaining epithelial barrier function and immune homeostasis.^10^ Its depletion has been shown to disrupt mitochondrial dynamics, impair tight junction integrity, and promote pro-inflammatory signaling, thereby exacerbating mucosal damage.^11^^,^^12^ Restoration of NAD^+^ levels can occur via three distinct routes: *de novo* synthesis from Trp via the KP, the salvage pathway (using nicotinamide [NAM] or nicotinamide riboside [NR]), and the Preiss–Handler pathway (using nicotinic acid [NA]).^13–17^ In several inflammatory diseases, similar patterns of KP activation accompanied by an unexpected NAD^+^ decline have been reported, and supplementation with NAD^+^ precursors can restore cellular energy homeostasis.^18–20^

Recent work by Minhas et al. identified a potential explanation for the apparent paradox: they showed that in macrophages, lipopolysaccharides (LPS; an acute inflammatory signal) suppress expression of *QPRT*, the enzyme converting QA to NAD^+^, thereby blocking *de novo* NAD^+^ synthesis despite upstream KP activation.^21^ However, it remains unclear whether this metabolic bottleneck is relevant in chronic inflammation, whether it also involves non-myeloid cells, or whether it directly impacts disease progression.

Here, we identify a similar bottleneck in *de novo* NAD^+^ synthesis in the context of IBD, driven by reduced *QPRT* expression. We show that *QPRT* is suppressed in the inflamed mucosa, resulting in QA accumulation and insufficient NAD^+^ production despite strong KP activation. This bottleneck is maintained by upstream activation of the JAK/STAT pathway, which enhances IDO1-mediated Trp degradation. In patient serum and dextran sodium sulfate (DSS)-induced colitis, QA levels are elevated, while mucosal NAD^+^ is depleted. *In vitro*, *QPRT* knockdown promotes inflammatory responses, which are reversed by NAD^+^ precursor supplementation. Targeting this bottleneck, either by restoring QPRT activity or bypassing QPRT reduction with salvage pathway substrates, represents a potential therapeutic approach to alleviate energy deficiency and inflammation in IBD.

## 2. Materials and methods

### 2.1. Patient consent

Clinical studies at University Hospital Schleswig-Holstein, Campus Kiel, were approved by the ethics committee of Kiel University (D 490/20, D 489/20, A 124/14, AZ 156/03-2/13, EA 1/300/15) following the *Declaration of Helsinki*. Enrolled subjects provided written informed consent. Procedures were carried out in accordance with national guidelines. Clinical characteristics are recorded in Tables S1–S3.

### 2.2. Targeted MS for Trp derivatives in human serum (cohorts #1 and #1a)

Samples were measured at Biocrates Life Sciences AG (Innsbruck, Austria) with their ‘Tryptophan Metabolism Assay’ via ultra-high-performance liquid chromatography mass spectrometry (HPLC-MS) with multiple reaction monitoring in positive mode using a SCIEX API 5500 QTRAP^®^ (AB SCIEX, Germany) and electrospray ionization. Half of measurements were performed after phenyl isothiocyanate derivatization. Analytes were quantified with external seven-point calibration.

Metabolites below the limit of quantitation and detection were considered missing; those with ≥80% non-missing values were retained (80% rule^22^). Following filtering, 3-OH-anthranilic acid (3OHAnth), quinaldic acid, Xanth and 3-IPA had missing values (1.2%, 2.9%, 2.3%, 6.1%). These values were imputed as half of each metabolite’s minimum value. Metabolites were log_10_ transformed and auto-scaled prior to statistical analysis.

The software MetaboINDICATOR^TM^ (Biocrates) was used to further reveal the biological significance of metabolic changes observed. In addition to the pre-calculated ratios provided, we included two ratios (QA:sumIDO and QA:3OHAnth) based on reported increases to these ratios in disease flares.^8^ QA:sumIDO was calculated as: QA/(3OHAnth + 3-OH-kynurenine [3OHKyn] + KA + kynurenine [Kyn] + picolinic acid [PA] + QA + Xanth). The QA:Trp ratio reflects a measure of overall QA synthesis relative to the entire Trp pool.

For cohort #1, serum Trp was assessed by HPLC for *n* = 180 patients with *n* = 812 observations.

Targeted metabolomics was conducted in subcohort #1a of cohort #1 (*n* = 52 Crohn’s didease [CD], *n* = 82 ulcerative colitis [UC], *n* = 343 observations) with available samples at baseline, week 2, and week 14 after advanced therapy induction.

We included the following potential confounding variables to linear mixed models (LMMs) used to assess Trp changes over the first 52 weeks of biologic therapy: biologic therapy, diagnosis (UC/CD), body mass index (BMI), age at baseline, recruitment cohort, and sex assigned at birth. For targeted metabolomics, LMMs included the covariates: sex at birth, age, BMI, biologic status (at time of observation), and, where appropriate, diagnosis. Spearman correlations were performed using week 14 observations, where, owing to treatment, we expected the widest variance in disease activity. All analyses were performed using R v.4.4.0.

### 2.3. Animal use and care, DSS colitis

DSS colitis was performed at Pennsylvania State University, and approved by IACUC (PROTO202202188). Male 8- to 12-week-old C57BL/6J mice (Jackson Laboratory) were acclimatized for 7 days in specific-pathogen-free (SPF) conditions, receiving laboratory diet 5010. Mice were single-housed and supplied with autoclaved water with/without 2.5% DSS (MP Biomedicals) for 5 days (*n* = 8 control/*n* = 12 DSS), followed by regular water. DSS-water was exchanged every other day, and consumption was monitored. Disease activity indices (DAIs; weight loss, stool consistency, rectal bleeding, behavior, activity, fur) were assessed daily.

For NAM supplementation, male C57BL/6J mice (>12 weeks, Charles River France) were held under SPF conditions (Institut Pasteur de Lille, France; #B59‐35009) according to governmental guidelines (#2010/63/UE; Décret 2013‐118) and animal ethics (protocol #05273.01). While acclimatizing for 1.5 months, animals received diet A4 (Scientific Animal Food and Engineering, France) and water *ad libitum*. Animals were exposed to 12-h light–dark cycles (7 am to 7 pm). For NAM supplementation, a Trp-free diet (Ssniff, Germany; powdered to prepare pellets) supplemented with control granules or a controlled NAM release formulation (Solural Pharma, France) in three different concentrations was provided for 12 days prior to DSS treatment (1.5%, TDB Consultancy, Sweden) for 5 days (*n* = 10 per group). The diet was continued throughout the experiment. DAIs (diarrhea, fecal blood, macroscopic appearance of inflammation) were obtained daily.

Experiments followed ARRIVE guidelines.

### 2.4. Integrative transcriptomics and metabolomics, metabolic modeling (cohort #2)

Blood and biopsies were obtained from two longitudinal IBD intervention cohorts from northern Germany (*n* = 32 UC, *n* = 30 CD) over 14 weeks. Patients who were treatment-naive for biologics were introduced to anti-TNFα (*n* = 22), anti-α4β7-integrin (*n* = 21), anti-IL6-trans-signaling (*n* = 16), or anti-IL6-R (*n* = 3) and were clinically and endoscopically monitored. In total, 120 biopsies and 123 whole blood samples were used for RNA sequencing (RNA-seq), and 128 serum samples for metabolomics (Biocrates MxP Quant 500 kit, Biocrates Life Sciences AG, Austria) as previously described.^3^^,^^23^^,^^24^ Analysis of differentially expressed genes (DEGs) was performed with variance stabilized read counts (DESeq2) and applying LMMs (lme4) (vst ∼ sex * HBI/Mayo|Kyn: Trp + [1 | Patient ID]). Enrichment analyses were based on means of gene set enrichment (GSEA) or hypergeometric tests for overrepresentation (ClusterProfiler). For GSEA, a ranking vector was created summing t-values for the main effect of HBI (Harvey–Bradshaw Index)/Mayo or serum Kyn:Trp and interaction with sex. Analyses were performed in R. All *P*-values were adjusted with Benjamini–Hochberg correction.

Trp degrading pathways were extracted with the human metabolic model (recon3D). Expression values (as transcripts per million [TPM]) were mapped to Trp pathways applying gene–protein–reaction relations of recon3D (github.com/Porthmeus/CORPSE) to estimate reaction activity scores (RAS). RAS were associated with HBI/Mayo scores in LMMs (rxnExpr ∼ sex * HBI/Mayo + [1 | Patient ID]). Linear modeling was used to impute missing HBI/Mayo according to individual time trajectories assuming a logarithmic decline in HBI/Mayo over time. All *P*-values were adjusted with Benjamini–Hochberg correction.

### 2.5. Correlation of mucosal gene expression with disease severity and ISG scores (cohort #3)

Mucosal expression of *IDO1* and *QPRT* were extracted from transcriptomics datasets GSE73661 (microarray of UC) and GSE109142 (RNA-seq TPM counts of pediatric UC) and processed using R v.4.2.2. Expression levels were correlated with (1) the Mayo endoscopic subscore for 166 biopsies from *n* = 67 UC (treated with infliximab or vedolizumab) and *n* = 12 HC (GSE7366125) patients, and (2) the histology severity score of *n* = 206 new-onset UC and *n* = 20 HC (GSE10914226) patients. *IDO1* and *QPRT* expression was furthermore correlated with IFNγ, TNFα, and IL23 scores using the R package *singscore*,^25–28^ the IFNγ gene set (*IFITM1*, *MX1*, *OAS3*, *IFIT1*, *IFI44L*, *IFI16*),^29^ and the TNF and IL23 gene sets described by Martínez et al.^28^.

### 2.6. *IDO1* and *QPRT* in intestinal biopsies from UC patients (cohorts #4–6)

Normalized *IDO1* and *QPRT* expression in UC patient biopsies was assessed in published microarray and RNA-seq data. Endoscopic improvement was defined as endoscopic Mayo (eMayo) score ≤1. Biopsies were collected within three longitudinal cohorts. (1) UC patients were treated with tofacitinib (baseline = 27, week 8 = 25).^30^ Raw sequencing reads were assessed with FastQC (v.0.11.9)^31^ and aligned to the human reference genome (GRCh38) using STAR aligner (v.2.7.10a).^32^ Gene-level read counts were obtained with featureCounts from the Subread package (v.2.0.1) using Gencode (v.38) for gene models.^33^ DEG analysis was performed with DESeq2 (v.1.44.0) in R (v.4.4.1)^34^ correcting for age, sex, prednisone, anti-TNF, vedolizumab, eMayo, response, and visit. (2-3) A prospective study of UC subjects with active colonic disease received either vedolizumab or infliximab^24^^,^^35^ (vedolizumab-treated: baseline = 9; week 2 = 7; week 6 = 8; week 14 = 8; infliximab-treated: baseline = 8; week 2 = 7; week 6 = 7; week 14 = 6). RNA-seq was performed. Raw gene expression counts were normalized using the DESeq2 R package^34^ v.1.40.2.

### 2.7. Statistical analysis (*in vitro/in vivo* experiments)

Statistical tests were selected considering data distribution and variance characteristics: for *in vitro* experiments, unpaired Student’s t-tests (two groups) and one-way ANOVA with Šidák correction (multiple groups) were used. For animal experiments, Mann–Whitney U (two groups) or Kruskal–Wallis and Dunn’s correction (multiple groups) were employed. A threshold of *P* < .05 was considered statistically significant. Statistical tests were performed with GraphPad PRISM software 10.

## 3. Results

### 3.1. Longitudinal rewiring of Trp metabolism indicates therapeutic efficacy in IBD

**Figure 1.**
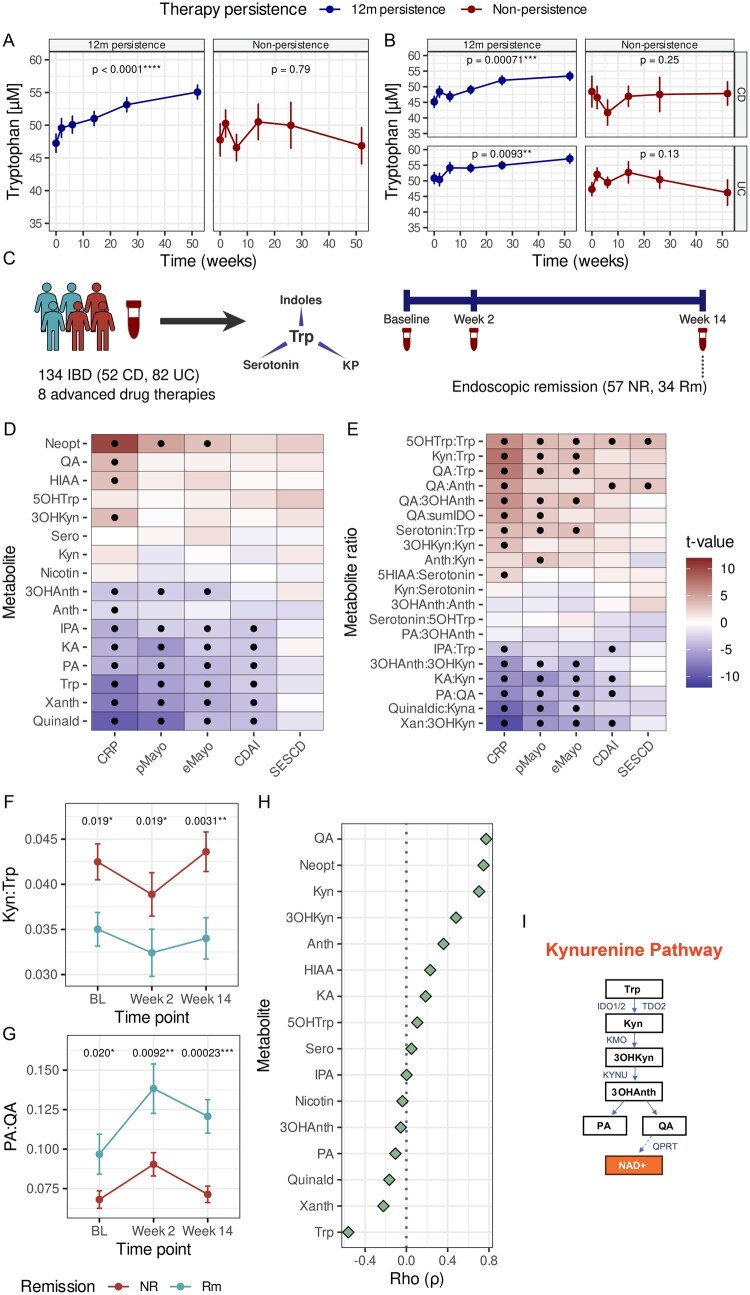
Longitudinal tryptophan (Trp) (derivative) trajectories indicate restored Trp metabolism with therapy success. (A, B) Longitudinal HPLC-measured Trp trajectories for IBD patients with 12-month persistence or discontinuation of biologic therapy. UC and CD are plotted together and separated by diagnosis (cohort #1). (C) Structure of cohort #1a for targeted Trp metabolomics. Endoscopic remission (week 14) based on eMayo (<2) or SES-CD (<3). (D, E) LMMs were used to identify links between Trp derivatives and the indicated disease activity metrics. Black dots indicate statistical significance (*P*_adj_ = .05; cohort #1a). (F, G) Selected Trp derivative trajectories in endoscopic remitters by week 14. LMMs were used to assess the difference, with contrast analysis applied to assess group interaction at each time point (cohort #1a). NR: non-remission, Rm: remission. (H) Spearman correlation of baseline Trp derivatives with Kyn:Trp ratio. (I) Simplified KP overview. Neopt: neopterin, HIAA: 5-hydroxy indoleacetic acid, 3OHKyn: 3-hydroxy-kynurenine, 5OHTrp: 5-hydroxy-tryptophan, Sero: serotonin, Nicotin: nicotinamide, Anth: anthranilic acid, 3OHAnth: 3-hydroxy-anthranilic acid, IPA: indole-3-propionic acid, KA: kynurenic acid, PA: picolinic acid, Xanth: xanthurenic acid, Quinald: quinaldehyde, QA: sumIDO: QA/(3-OHAnth + 3-OHKyn + KA+Kyn + PA+QA + Xanth). Created with Biorender.com.

Patients achieving long-term disease control (*n* = 120) exhibited a stable and continuous rise of serum Trp over the entire 52 weeks (Figure 1A), independent of disease entity (UC vs. CD, Figure 1B; cohort #1, Table S1). By contrast, patients without therapy persistence after 1 year (*n* = 60) did not exhibit a rise in serum Trp concentration (Figure 1A, B). We conducted targeted metabolomics in a subcohort of cohort #1 to examine longitudinal changes of Trp and corresponding KP, indolic, and serotonin metabolites (Fig. S1) in IBD patients (*n* = 52 CD, *n* = 82 UC) with available samples at baseline, week 2, and week 14 after advanced therapy induction (cohort #1a; Figure 1C, Table S2). Of these, 91 patients with available endoscopic scores at week 14 were classified into remission and non-remission based on eMayo (<2) or simple endoscopic score of CD (SES-CD; <3; *n* = 34 remission, *n* = 57 non-remission). We found significant differences in the second principal component between remission and non-remission (Figure S2A), whereas baseline inflammation levels were comparable between remitters and non-remitters (Figure S2B).

We assessed individual associations between metabolites or metabolite ratios and clinical (Crohn’s disease activity index: CDAI, CD; partial Mayo: pMayo, UC), endoscopic (SES-CD, CD; eMayo, UC), and biochemical (C-reactive protein: CRP) disease activity indices (Figure 1D, E), to identify serum metabolites with temporal fluctuations coinciding with inflammation resolution (defined as change from active disease to endoscopic remission). In line with previous studies, the Kyn to Trp (Kyn:Trp) ratio was higher in individuals not achieving remission at week 14 at weeks 0, 2 and 14 (Figure 1F).^3^^,^^7^^,^^8^ The ratio of PA to QA (PA:QA) was higher in remitters at each time point tested (Figure 1G). Additionally, the correlation between QA and Kyn:Trp was stronger for QA (Spearman’s rho, ρ = 0.77, false discovery rate [FDR]-corrected *P* < .0001) compared to PA (ρ = −0.11, FDR-corrected *P* = .38) (Figure 1H). These data show that Trp degradation along the KP resulting in QA accumulation (Figure 1I) is associated with disease activity and an unfavorable disease course in IBD.

### 3.2. Transcriptome-aided metabolic modeling reveals metabolic constraint at QA-to-NAD^+^ conversion in the inflamed mucosa

**Figure 2.**
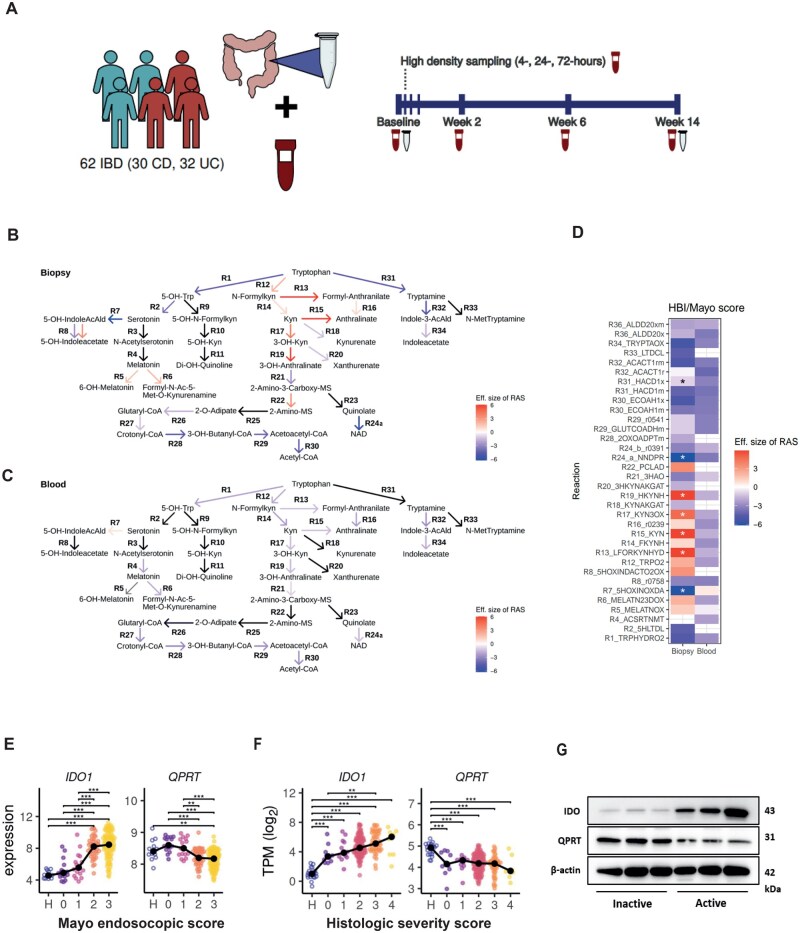
Transcriptome-aided metabolic modeling reveals mucosal block at QA-to-NAD^+^ conversion. (A) Whole blood and biopsies were sampled within two longitudinal IBD intervention cohorts (*n* = 32 UC, *n* = 30 CD; cohort #2). (B, C) Trp degrading pathways were extracted with the human metabolic model (recon3D). Gene expression was mapped to Trp degradation by applying gene–protein–reaction relations of recon3D to estimate reaction activity scores (RAS). RAS were associated with HBI/total Mayo in LMMs. Model effect sizes (t-value) are color-coded in arrows (black: missing data due to pre-filtering/missing gene-to-reaction rule). (D) Effect sizes (t-value) of the LMMs employed in (C) are displayed as a heatmap. All *P*-values were adjusted with the Benjamini–Hochberg correction. (E, F) Expression of *IDO1* and *QPRT* is shown in association with (1) endoscopic disease activity in 166 biopsies (*n* = 67 UC, *n* = 12 HC) (GSE7366125), (2) the histology severity score (*n* = 206 new-onset UC, *n* = 20 HC) (GSE10914226; cohort #3). Statistical analysis was performed with one-way-ANOVA (Tukey’s HSD post-hoc test). Black dots and connecting lines show median expression. (G) IDO1 and QPRT protein expression are displayed in human biopsies (inactive [eMayo/Nancy 0], active [eMayo 3/Nancy 4], *n* = 3 biological replicates per group; cohort #1a). **P < .01; ***P < .001. Created with Biorender.com.

We aimed to understand the underlying transcriptional changes in the inflamed mucosa driving QA accumulation in IBD patients’ serum using a second longitudinal IBD cohort (UC, *n* = 30; CD, *n* = 32) with serial biosampling before and after advanced therapy induction (cohort #2; Figure 2A, Table S3).^23^^,^^24^^,^^35^ By estimating reaction activity scores of enzymatic reactions catabolizing Trp in bulk transcriptomics from blood and intestinal mucosa, we correlated reaction abundances of Trp-degrading pathways with clinical disease activity indices (HBI, CD; total Mayo score, UC).^36^^,^^37^ We identified significant correlations of Trp-degrading enzymes with disease activity in the mucosa, but not in the blood (Figure 2B–D). Inferring mucosal flux of Trp metabolism, we observed decreased reaction abundance for genes converting QA into nicotinic acid mononucleotide (NAMN) (reaction ID R24a) upon high disease activity, indicating serum QA accumulation might result from insufficient mucosal conversion into NAD^+^ (Figure 2B, D). To confirm this hypothesis, we assessed *IDO1* and *QPRT* expression in the inflamed colonic mucosa. *IDO1* and *QPRT* in transcriptomics from sigmoid UC biopsies significantly correlated with endoscopic (Figure 2E) and histological (Figure 2F) disease activity (cohort #3).^38^^,^^39^ Whereas *IDO1* expression increased with inflammatory severity, *QPRT* decreased (Figure 2E, F). Using intestinal UC biopsies (eMayo 0 vs. 3; cohort #1a), we confirmed heightened protein expression of IDO1 and reduced QPRT with enhanced mucosal inflammation (Figure 2G).

**Figure 3.**
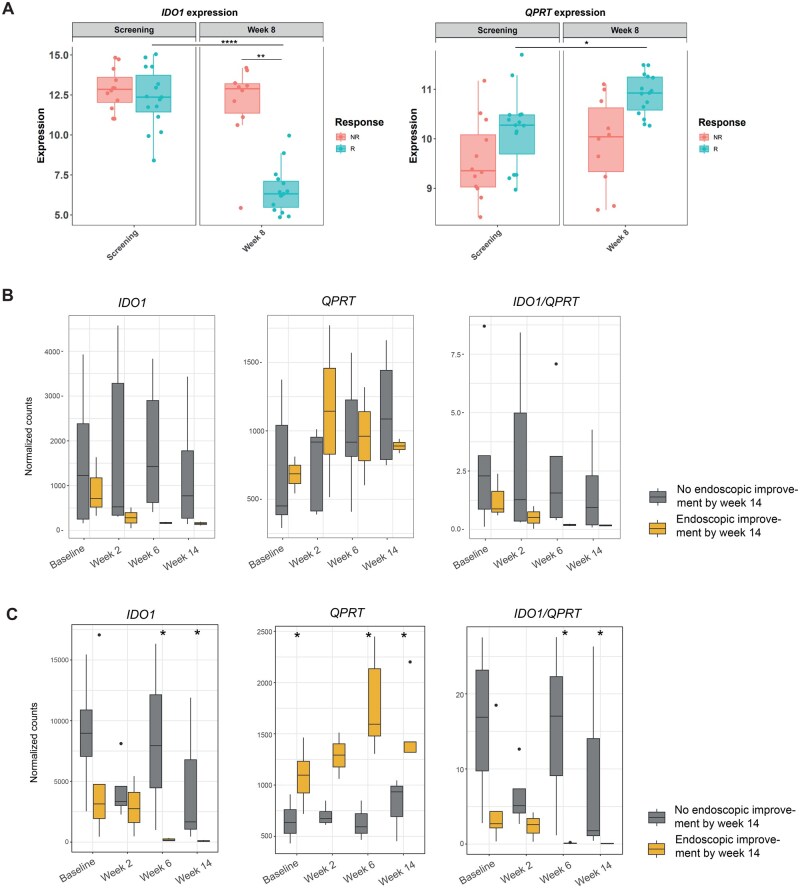
Mucosal *IDO1* and *QPRT* expression reflect therapy response. (A) Expression of *IDO1* and *QPRT* within mucosal transcriptomes between endoscopic responders (15) and non-responders (12) in tofacitinib-treated UC (cohort #4).^32^ Endoscopic improvement defined as Mayo endoscopic subscore ≤1. (B, C) The normalized expression of *IDO1*, *QPRT*, and *IDO1*/*QPRT* ratio in mucosal biopsies of UC patients was assessed for different biological treatments. Endoscopic improvement was defined as a Mayo endoscopic subscore ≤1. (B) UC patients were treated with anti-TNFα (infliximab) within a longitudinal IBD cohort study. A total of 28 longitudinal samples were included in the analysis (baseline = 8; week 2 = 7; week 6 = 7; week 14 = 6; cohort #5). (C) UC patients were treated with anti-α4β7-integrin (vedolizumab) within a longitudinal IBD cohort study. A total of 32 longitudinal samples were included in the analysis (baseline = 9; week 2 = 7; week 6 = 8; week 14 = 8; cohort #6). For (B, C), the Wilcoxon test was used to determine significant differences between responders and non-responders at each time point. *P < .05; **P < .01; ***P < .0001. Created with Biorender.com.

We next assessed whether mucosal *IDO1* and *QPRT* expression is also informative of the response towards advanced therapies. We therefore analyzed mucosal transcriptomics of UC patients treated with the JAK inhibitor (JAKi) tofacitinib (cohort #4),^30^ infliximab or vedolizumab (Figure 3; cohort #5/6). Regardless of the therapeutic class, *IDO1* was downregulated and *QPRT* upregulated between baseline and follow-up in endoscopic responders, resulting in overall lower mucosal *IDO1* and heightened *QPRT* levels in responders vs. non-responders at follow-up. This pattern suggested that resolution of inflammation restores mucosal *IDO1* and *QPRT* expression.

### 3.3. DSS colitis confirms a mucosal metabolic constraint at QA

**Figure 4.**
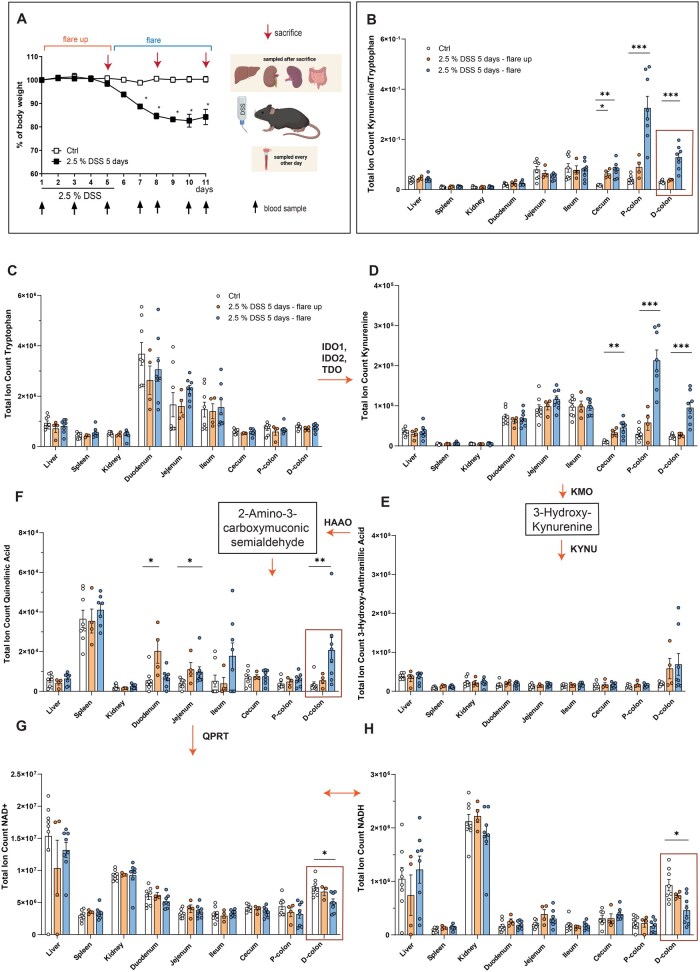
DSS colitis validates mucosal metabolic constraint at QA. (A) Male C57BL/6J mice (*n* = 20) received water (white, control) or 2.5% DSS for 5 days (black, water from d6). Blood was collected every other day (black arrows). Animals were killed (*n* = 4 control/DSS) on d5/8/11 (red arrows). (B–H) Total ion counts were measured by LC-MS in the indicated tissues. White = control groups (killed d5/8, *n* = 8); orange: colitis flare-up (killed d5, *n* = 4), blue: flare (killed d8/11, *n* = 8). Each dot represents one mouse. Data presented as mean ± SEM. Statistical analysis was performed with Kruskal-Wallis. *P < .05; **P < .01; ***P < .001; ****P < .0001. D/P-Colon: distal/proximal colon. Created with Biorender.com.

To confirm our findings experimentally and to show that mucosal QA accumulation coincides with NAD^+^ depletion, we used DSS colitis for targeted metabolomics of serum and tissues from different inflammation states (*flare-up* [day 5; d5], *flare* [d8/11]) (Figure 4A). Starting from d3, serum Kyn increased, with heightened Kyn:Trp ratios from d5 to d11 (Figure S5). When analyzing Trp and derivatives in all anatomical locations *post mortem*, we found that Trp degradation almost exclusively occurred in the inflamed colon (Figure 4B, D). Trp catabolism coincided with mucosal QA accumulation and reduced NAD(H), linking KP activation with NAD^+^ depletion in inflammation (Figure 4F–H). Therefore, our data support mucosal QA build-up and decreased NAD(H) as key metabolic changes during mucosal inflammation.

### 3.4. Mucosal Trp catabolism in IBD is regulated by JAK/STAT and NF-κB signaling

**Figure 5.**
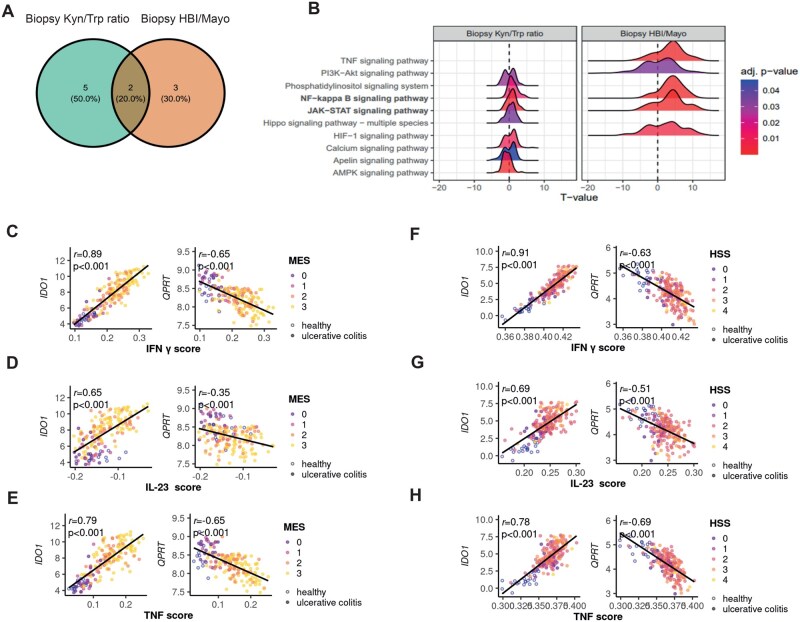
Mucosal Trp catabolism in IBD is regulated by JAK/STAT and NF-κB signaling. (A, B) Longitudinal sampling within two longitudinal IBD intervention cohorts (*n* = 32 UC, *n* = 30 CD; cohort #2). Transcriptomics was conducted on whole blood and biopsies; serum was used for metabolomics. DEG analysis was performed applying LMMs to associate gene expression changes with changes in HBI/total Mayo and serum Kyn/Trp ratios. Gene set enrichment and hypergeometric tests were employed on signal transduction pathways annotated by KEGG. All *P*-values were adjusted with the Benjamini-Hochberg correction. (C–H) For Figure 2E, F, the putative upstream cytokine network was inferred, *r* = Pearson’s correlation coefficients. *P*-values are FDR-corrected. Created with Biorender.com.

Having shown that Trp degradation in the inflamed mucosa leads to accumulation of QA and depletion of NAD^+^, we aimed to pinpoint upstream transcriptional signatures driving Trp catabolism in IBD that feed into the metabolic constraint at QPRT. Therefore, we combined bulk transcriptomics of blood and intestinal biopsies with serum metabolomics (cohort #2, longitudinal sampling from UC, *n* = 30; CD, *n* = 32; Figure 2A) to integrate expression of Trp-degrading enzymes with serum Trp catabolism. We identified commonly regulated signal transduction pathways correlating with (1) serum Kyn:Trp ratios as a proxy for Trp degradation and (2) disease activity scores (HBI/total Mayo) using LMMs. Among the pathways found for both approaches, nuclear factor ‘kappa-light-chain-enhancer’ of activated B-cells (NF-κB) and JAK/STAT were upregulated in inflamed tissue (Figure 5A, B). Assessing overlapping signaling transduction pathways between blood and tissue in association with high disease activity and elevated serum Kyn:Trp, we validated upregulation of mucosal JAK/STAT signaling (Figure S6A, B).

We confirmed the molecular link between JAK/STAT signaling and mucosal expression of the key KP enzyme *IDO1*, a known JAK/STAT downstream target gene, by inferring the putative upstream cytokine network (cohort #3).^40^^,^^41^ Mucosal *IDO1* induction significantly correlated with IFNγ, IL23, and TNF, implying mucosal JAK/STAT-dependent cytokines drive KP-mediated Trp breakdown (Figure 5C–H). Interestingly, we noted a similar association between JAK/STAT signaling and QPRT downregulation, suggesting that IDO1 upregulation and QPRT suppression coincide upon inflammation (Figure 5C–H).

### 3.5. JAK inhibition abrogates Trp catabolism and QA accumulation

**Figure 6.**
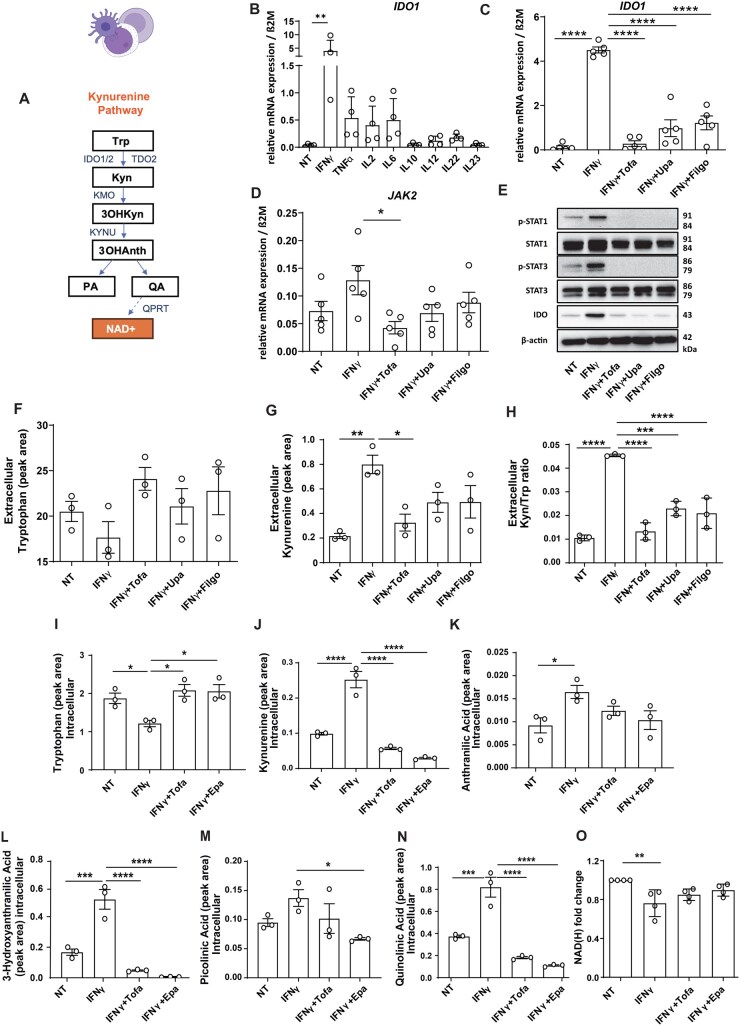
Blockade of the KP by JAK inhibition abrogates QA accumulation. (A) KP overview. (B) RT-qPCR of human PBMCs were stimulated with 10 ng/mL of the indicated cytokines for 24 h (*n* = 2 technical/*n* = 4 biological replicates). (C–H) Human PBMCs were incubated with IFNγ (10 ng/mL) ± 1 µm tofacitinib (Tofa), 0.1 µm upadacitinib (Upa) or 10 µm filgotinib (Filgo) for 24 h. (C, D) RT-qPCR (*n* = 2 technical/*n* = 5 biological replicates), housekeeping gene: β2 microglobulin (β2M). (E) (phospho)STAT1/3 and IDO1 were normalized to β-actin (*n* = 3 biological replicates). (F–H) Extracellular Trp and Kyn were measured by LC-MS (*n* = 3 technical replicates/*n* = 3 biological replicates; intracellular metabolites: Figure S7C–E). (I–O) Human PBMCs were incubated with IFNγ (10 ng/mL) ± Tofa (1 µm) or epacadostat (Epa, 5 µm) for 24 h. LC-MS of cell lysates (*n* = 3 technical/*n* = 3 biological replicates; extracellular metabolites: Figure S8B–F). Metabolite abundances were normalized to cell numbers. (O) NAD/NADH-Glo^TM^ assay on the experimental set-up of (I–N) (*n* = 2 technical/*n* = 4 biological replicates). Data presented as mean ± SEM. Statistical analysis with one-way-ANOVA. *P < .05; **P < .01; ***P < .001, ****P < .0001. Created with Biorender.com.

We hypothesized that JAK/STAT-mediated activation of the KP results in QA accumulation. Given the known robust expression of *IDO1* in immune cells, peripheral blood mononuclear cells (PBMCs) were chosen as an *in vitro* model to investigate Trp catabolism along the KP.^42^ After identifying IFNγ as the strongest *IDO*-inductor in human PBMCs (Figure 6B, Figure S7A), we co-treated PBMCs with IFNγ and JAKi (pan-JAKi: tofacitinib; selective JAKi: upadacitinib, filgotinib). IFNγ-induced IDO gene and protein expression was completely blocked upon co-treatment with JAKi (Figure 6C–E, Figure S7B). JAK/STAT-activated IDO1 upon IFNγ stimulation enhanced Trp-to-Kyn turnover in PBMC lysates and supernatants, which was reverted by JAKi (Figure 6F–H, Figure S7C–E). To show elevated Kyn results from Trp degradation and no other potential sources, we employed isotope-labeled Trp (^13^C_11_ Trp) in PBMCs. Again, IFNγ induced conversion of ^13^C_11_ Trp into ^13^C_10_ Kyn, which was abrogated by JAKi (Figure S7F–K). To investigate whether JAK/STAT-driven activation of the KP led to QA accumulation, we profiled downstream Trp metabolites by subjecting PBMCs to IFNγ ± tofacitinib or epacadostat (IDO1 inhibitor). IFNγ induced increases of Kyn and the KP metabolites anthranilic acid (Anth), 3OHAnth, and QA (Figure 6I–N, Figure S8B–F). Conversely, coadministration of tofacitinib or epacadostat abrogated QA accumulation. QA was undetectable in supernatants, suggesting intracellular accumulation (Figure S8B–F).

We aimed to further validate that IFNγ-induced Trp catabolism along the KP resulted in QA accumulation in PBMC lysates due to a KP blockade at QPRT. Indeed, *QPRT* expression was suppressed following KP activation with IFNγ treatment (Figure S8G). We speculated that reduced *QPRT* not only caused QA accumulation, but also decreased QA-to-NAD^+^ conversion. NAD(H) production as assessed by an NAD/NADH-Glo^TM^ assay in human PBMCs was indeed diminished by IFNγ (Figure 6O), which was further aggravated in Trp-deficient medium, confirming Trp as the major source of NAD^+^ synthesis upon JAK/STAT activation (Figure S8H). Together, our data show that JAK/STAT-mediated Trp degradation via the KP feeds into QA accumulation due to a blockade of *de novo* NAD^+^ synthesis at QPRT.

### 3.6. QPRT modulates cellular inflammation by provision of NAD^+^

Lastly, we aimed to understand the direct impact of QPRT on modulating immune responses in the three most relevant intestinal cell types, namely PBMCs, fibroblasts, and intestinal epithelial cells (IECs).

**Figure 7.**
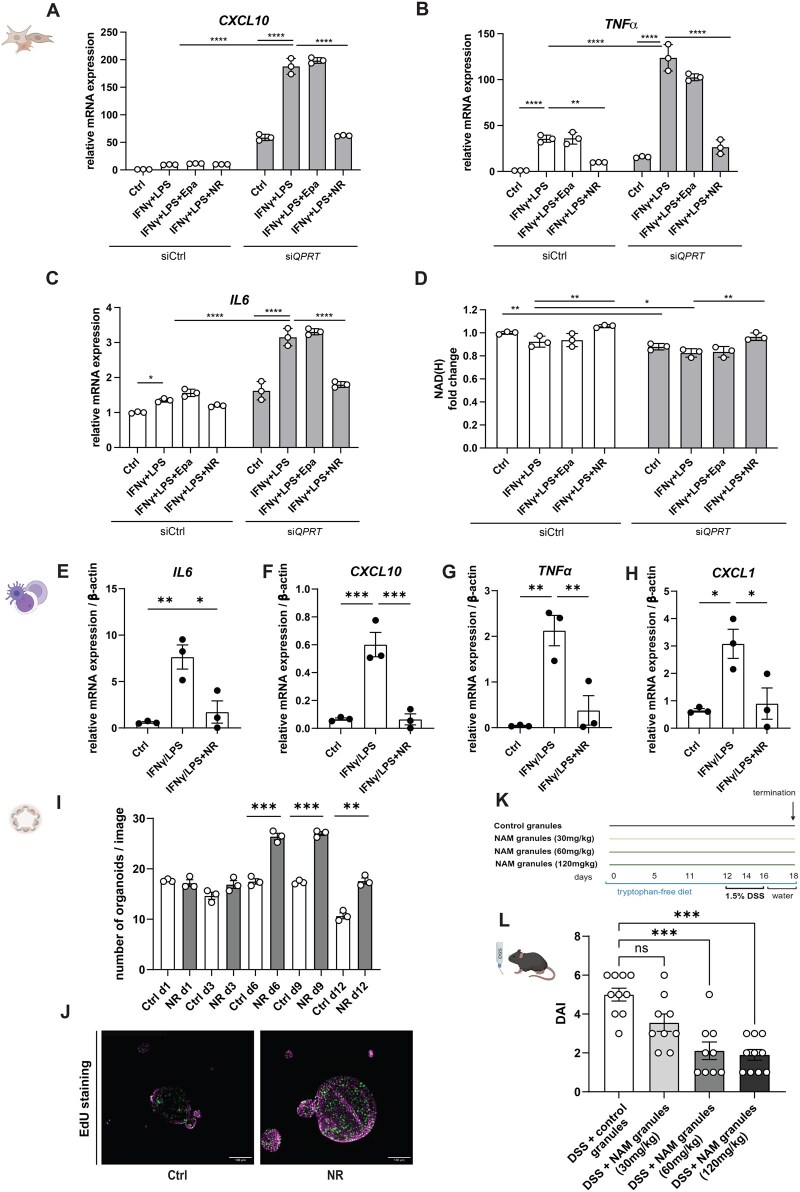
QPRT modulates cellular inflammation by provision of NAD^+^. Human fibroblasts were stimulated with IFNγ/LPS (1 µg/mL, 2 h) ± tofacitinib (Tofa, 1 µm), epacadostat (Epa, 5 µm) or nicotinamide riboside (NR, 10 mm) for 24 h. *QPRT* was silenced with siRNA. (A–C) RT-qPCR, housekeeping gene: β-actin. (D) NAD/NADH-Glo^TM^ assay (*n* = 3 technical/*n* = 3 biological replicates). (E–H) Human PBMCs were cultivated in Trp-deficient medium and treated with IFNγ (10 ng/mL, 22 h)/LPS (100 ng/mL, 2 h) ± NR (3 mm, 22 h). RT-qPCR, housekeeping gene: β-actin. Statistical analysis was done with one-way ANOVA (*n* = 3 technical/*n* = 3 biological replicates). (I) Number of human UC colonoids (eMayo 2) counted on indicated days post-seeding (NR 10 mm, started 24 h post-seeding). Statistics were performed with unpaired Student’s t-test. (J) Representative images of stainings (EdU: green, DAPI: purple) of human colonoids (Ctrl vs. NR-treated) (*n* = 3 technical replicates; *n* = 2 males, *n* = 1 female). (K) DSS colitis overview: male C57BL/6J mice were fed a Trp-free diet with control/NAM-containing granules for 12 days before treatment with 1.5% DSS for 5 days (*n* = 10 per group). (L) DAI on d18. Data presented as mean ± SEM, statistical analysis with Kruskal–Wallis test. *P < 0.05; *P < 0.01; ***P < 0.001; ****P < 0.0001. Created with Biorender.com.

To silence *QPRT*, we utilized siRNA in fibroblasts and IECs, whereas for PBMCs, we embarked on our previous finding of downregulated *QPRT* following IFNγ treatment (Figure S8G). *QPRT* suppression significantly increased pro-inflammatory cytokines upon stimulation with IFNγ and LPS (Figure 7A–H, Figure S9A–F).

To clarify whether NAD^+^ depletion or QA buildup drives cytokine expression in *QPRT*-silenced cells, we tested QA’s cytotoxic effects. QA neither amplified cytokine responses nor significantly reduced viability of PBMCs (Figure S10A–D), IECs (Figure S10E–H), or fibroblasts (Figure S10I–K), arguing against direct pro-inflammatory effects of QA.

We thus hypothesized that *QPRT* knockdown drives pro-inflammatory cytokine expression via impaired *de novo* NAD^+^ synthesis and assessed cytokine expression and NAD(H) levels following treatment with IFNγ and LPS in combination with the NAD^+^ precursor NR.^43^ NR supplementation ameliorated increased cytokine induction in *QPRT*-silenced fibroblasts, pointing towards a protective role of restoring cellular NAD(H) levels (Figure 7A–C). Indeed, NAD(H) levels were significantly reduced upon *QPRT* knockdown, which was further aggravated by concomitant IFNγ and LPS stimulation and rescued by NR supplementation (Figure 7D, Figure S9G). Notably, the IFNγ- and LPS-induced increase of pro-inflammatory cytokines in *QPRT*-silenced cells was not ameliorated by epacadostat, again arguing against QA as the main inflammation driver (Figure 7A–C). Further confirming that NR can bypass insufficient *de novo* NAD^+^ synthesis from Trp, the anti-inflammatory property of NR was validated in IFNγ- and LPS-treated PBMCs under Trp-free conditions (Figure 7E–H). Similarly, NR treatment of human organoids derived from moderately inflamed colon of UC patients significantly boosted their growth (Figure 7I, J).

Lastly, we assessed if supplementation with NAD^+^ precursors would similarly lessen intestinal inflammation *in vivo*. To induce robust abrogation of *de novo* NAD^+^ synthesis from Trp via QPRT, DSS colitis was conducted in C57BL/6J mice that were fed a Trp-free diet and treated with both control and NAM-containing granules prior to and throughout the DSS colitis (Figure 7K, L). Although it must be considered that the effects of NAM may be amplified due to pronounced mucosal shortage of NAD^+^ upon both inflammatory and artificial Trp-deficient conditions, NAM supplementation significantly reduced disease activity indices in a dose-dependent manner.

## 4. Discussion

Increased Trp degradation via the KP is a hallmark of inflammation across multiple immune-mediated diseases. Despite this pathway fueling *de novo* NAD^+^ synthesis, mucosal NAD^+^ deficiency is observed in IBD.^12^^,^^44^ Minhas et al. recently demonstrated that inflammatory signaling, such as via LPS, can impair NAD^+^ synthesis by suppressing QPRT, the enzyme converting QA to NAD^+^.^21^ Their observations suggest that inflammation may both induce Trp catabolism and constrain its completion.

Our findings extend this model of constrained *de novo* NAD^+^ synthesis to IBD. In patients undergoing successful therapy, serum Trp rises over time, consistent with reduced KP activity. We confirm prior associations between QA and disease activity and expand these findings by demonstrating that serum QA also correlates with Kyn/Trp ratios, suggesting KP activation induces a downstream metabolic blockage.^7^^,^^8^ This is supported by mucosal transcriptomics revealing decreased *QPRT* expression with increased disease activity in IBD, indicating that reduced *QPRT* contributes to NAD^+^ deficiency despite upstream shuttling of Trp into the KP. Of note, elevated QA levels are also observed in rheumatoid arthritis and neurodegenerative diseases, suggesting this bottleneck may be a broader feature of chronic inflammation.^7^^,^^45^^,^^46^

Examining mucosal and blood transcriptomes of IBD patients, we found that KP activity is largely confined to the inflamed mucosa. There, *IDO1* was strongly upregulated, while *QPRT* was downregulated in association with disease activity, suggesting the bottleneck in *de novo* NAD^+^ synthesis is spatially constrained. To confirm the spatial origin of QA accumulation and downstream NAD^+^ depletion, we performed targeted metabolomics of immunoactive anatomical compartments in DSS colitis. We validated that KP-mediated Trp degradation occurs almost exclusively at the inflamed site, where it coincides with elevated QA and reduced NAD(H), mirroring the human data. Targeted metabolomics of human biopsy samples will provide complementary validation. Together, these findings confirm that impaired *de novo* NAD^+^ biosynthesis at the inflammation site contributes to local energy deficiency and may perpetuate disease.

By integrating transcriptomics with metabolomics, we identified JAK/STAT signaling as one of the dominant pathways associated with both elevated Kyn/Trp and disease severity. We demonstrated that IFNγ and other cytokines signaling via JAK/STAT induced *IDO1* in human PBMCs; however, paradoxically KP activation did not fuel NAD^+^  *de novo* synthesis due to reduced QPRT. While our data demonstrate coinciding KP induction and *QPRT* suppression, suggesting simultaneous regulation of *IDO1* and *QPRT* by inflammatory cytokines, further work is required to elucidate the specific factors that directly mediate *QPRT* downregulation. However, several JAK/STAT-activating cytokines have been shown to induce a metabolic switch towards the NAD^+^ salvage to replenish NAD^+^, which constitutes a more energy-efficient route to maintain NAD^+^ levels as compared to *de novo* synthesis.^47^^,^^48^ In agreement with this and the established involvement of JAK/STAT signaling in intestinal inflammation, isotope tracing of Trp and NAM in acute DSS colitis revealed inflammation-driven systemic reprogramming toward the salvage pathway to counteract reduced *de novo* synthesis from Trp.^49^ JAK inhibitors are established therapies in several chronic inflammatory disorders, including IBD, which restore cellular ATP levels and mitochondrial function.^50–56^ In our study, while UC patients with normalized *IDO1* and *QPRT* expression at week 8 showed improved response to tofacitinib, similar effects were seen in infliximab- and vedolizumab-treated patients, indicating relief of the bottleneck at QPRT upon suppression of upstream inflammatory signals. However, it needs to be acknowledged that alleviation of inflammatory states by JAKi is also attributable to other mechanisms, such as modulation of cytokine networks, altered immune-cell activation, or effects on epithelial barrier function.^57^

QA accumulation has been linked to rheumatoid arthritis and neurological diseases as well as to oxidative stress, ATP exhaustion, and mitochondrial dysfunction;^45^^,^^46^^,^^58^ however, these studies did not assess whether impaired NAD^+^ synthesis contributes to QA buildup. In our model, QA alone did not provoke a pro-inflammatory response *in vitro*. Instead, NAD^+^ depletion, induced by *QPRT*-silencing, amplified cytokine production following IFNγ/LPS stimulation. This phenotype was rescued by NR but not by IDO1 inhibition, which also depletes NAD^+^. Our data thus suggest that the pro-inflammatory effects of the metabolic bottleneck at QPRT arise due to NAD^+^ exhaustion rather than QA toxicity. Nevertheless, it must be considered that knockdown of QPRT may elicit additional pro-inflammatory responses that were not assessed in the present study. Given that NAD^+^ repletion can improve mitochondrial function and cellular fitness,^59^ insufficient provision of NAD^+^ via QPRT might impact stem cell function and wound repair in intestinal inflammation.

As it has previously been described that microbiota mediate intestinal homeostasis upon mitochondrial perturbations, a limitation of the present study is that the impact of the intestinal microbiota on inflammation-associated metabolism has not yet been assessed in IBD patients.^60^ However, we have recently conducted microbial depletion in DSS colitis, revealing an essential role of the gut microbiota in regulating host Trp bioavailability and utilization, ultimately licensing host NAD^+^ metabolism.^61^

Together with evidence that NAD^+^ repletion improves epithelial growth and ameliorates colitis *in vivo*, our data highlight a previously underappreciated metabolic checkpoint in *de novo* NAD^+^ synthesis linking chronic inflammation to energy imbalance. Therapeutic strategies that restore local NAD^+^ via targeted delivery of precursors (NCT05258474, NCT06488625) or modulation of upstream inflammatory signals may provide benefit not only in IBD, but also in other chronic inflammatory conditions.

## Supplementary Material

jjag043_Supplementary_Data

## Data Availability

Data and analytic methods will be available to other researchers either in the materials and methods section or via publicly available online databases.
