# Supplementary material for: A metabolic constraint in de novo NAD+ synthesis drives mucosal inflammation in IBD
Source: J Crohns Colitis. 2026 Jun 1;20(5):jjag043. doi: 10.1093/ecco-jcc/jjag043 (PMC13223750; doi:10.1093/ecco-jcc/jjag043)
Supplement: jjag043_Supplementary_Data [file jjag043_supplementary_data.pdf]

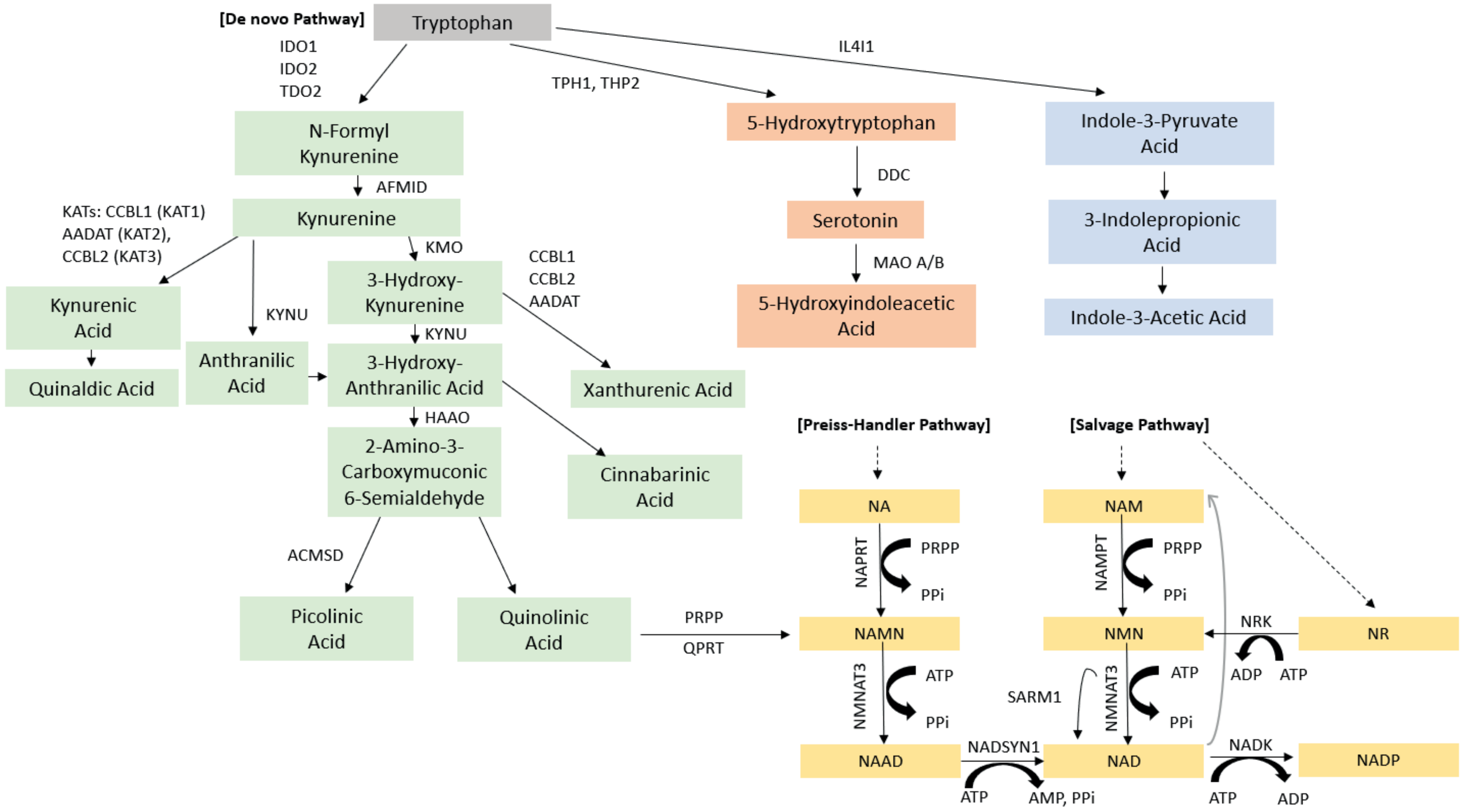

[De novo Pathway]
Tryptophan
IDO1
IDO2
TDO2
TPH1, THP2
IL4I1
N-Formyl Kynurenine
AFMID
Kynurenine
KATs: CCBL1 (KAT1)
AADAT (KAT2),
CCBL2 (KAT3)
KMO
KYNU
Kynurenic Acid
Quinaldic Acid
Anthranilic Acid
3-Hydroxy-Kynurenine
KYNU
3-Hydroxy-Anthranilic Acid
CCBL1
CCBL2
AADAT
Xanthurenic Acid
HAAO
2-Amino-3-Carboxymuconic 6-Semialdehyde
Cinnabarinic Acid
ACMSD
Picolinic Acid
Quinolinic Acid
PRPP
QPRT
5-Hydroxytryptophan
DDC
Serotonin
MAO A/B
5-Hydroxyindoleacetic Acid
Indole-3-Pyruvate Acid
3-Indolepropionic Acid
Indole-3-Acetic Acid
[Preiss-Handler Pathway]
[Salvage Pathway]
NA
NAPRT
PRPP
PPi
NAMN
NMNAT3
ATP
PPi
NAAD
NADSYN1
ATP
AMP, PPi
NAM
NAMPT
PRPP
PPi
NMN
NMNAT3
ATP
PPi
SARM1
NAD
NRK
ADP
ATP
NR
NADK
ATP
ADP
NADP

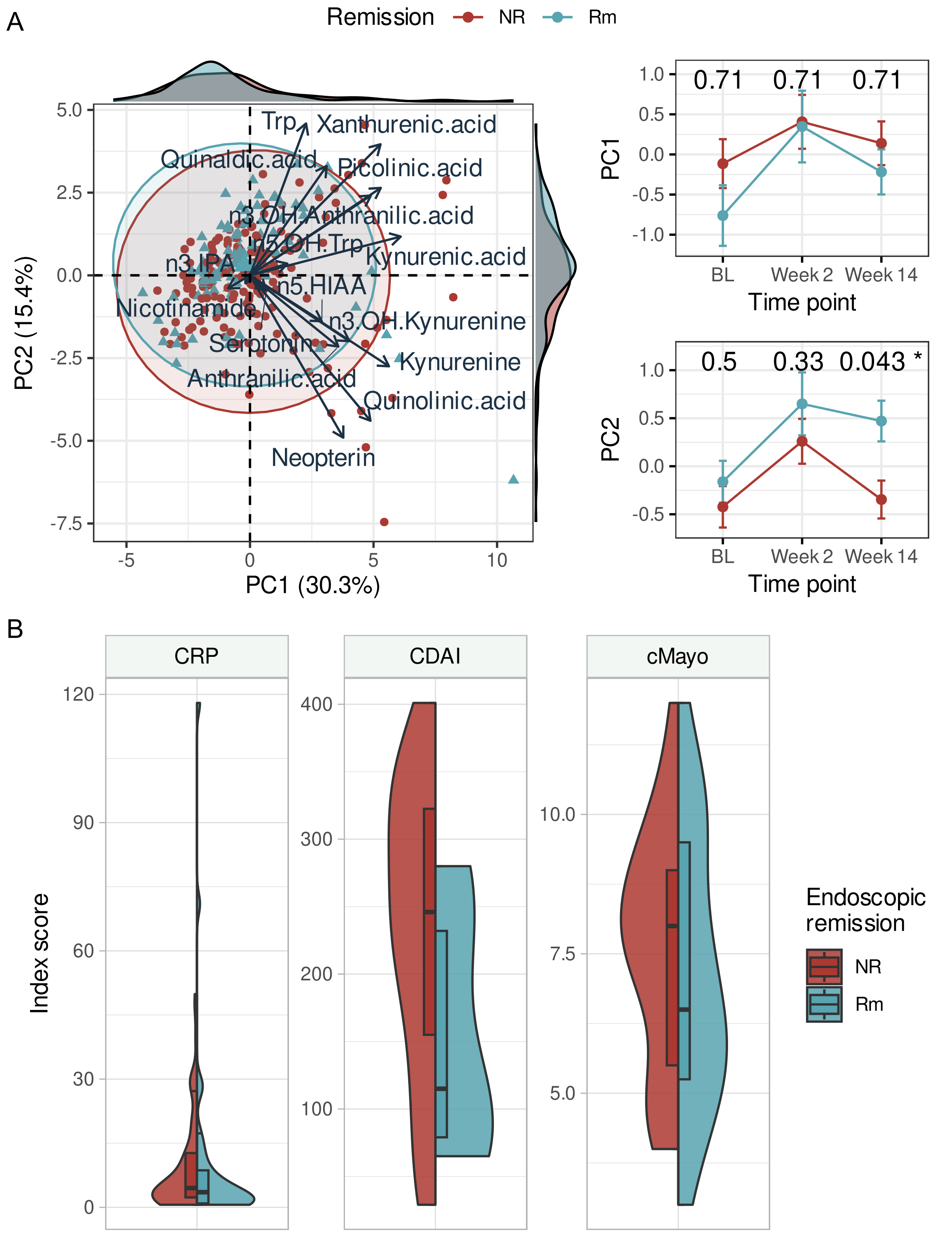

A
Remission
NR
Rm
Trp
Xanthurenic.acid
Quinaldic.acid
Picolinic.acid
n3.OH.Anthranilic.acid
n5.OH.Trp
Kynurenic.acid
n3.IPA
n5.HIAA
Nicotinamide
n3.OH.Kynurenine
Serotonin
Kynurenine
Anthranilic.acid
Quinolinic.acid
Neopterin
PC2 (15.4%)
PC1 (30.3%)
0.71 0.71 0.71
PC1
Time point
BL Week 2 Week 14
0.5 0.33 0.043 *
PC2
B
CRP
CDAI
cMayo
Index score
Endoscopic remission
NR
Rm

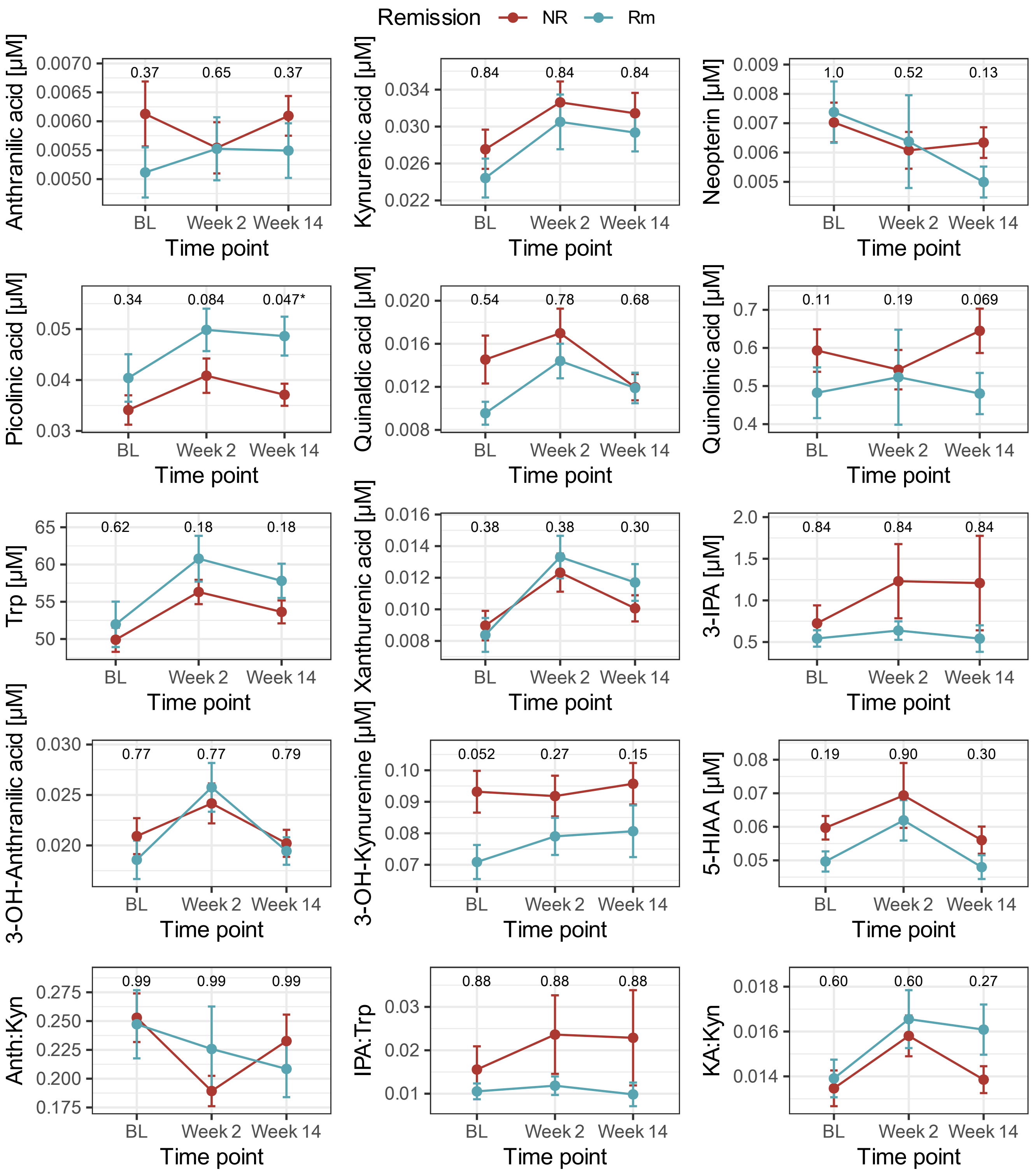
Remission NR Rm
Anthranilic acid [μM]
Kynurenic acid [μM]
Neopterin [μM]
Picolinic acid [μM]
Quinaldic acid [μM]
Quinolinic acid [μM]
Trp [μM]
Xanthurenic acid [μM]
3-IPA [μM]
3-OH-Anthranilic acid [μM]
3-OH-Kynurenine [μM]
5-HIAA [μM]
Anth:Kyn
IPA:Trp
KA:Kyn
Time point
BL Week 2 Week 14
0.37 0.65 0.37
0.84 0.84 0.84
1.0 0.52 0.13
0.34 0.084 0.047*
0.54 0.78 0.68
0.11 0.19 0.069
0.62 0.18 0.18
0.38 0.38 0.30
0.84 0.84 0.84
0.77 0.77 0.79
0.052 0.27 0.15
0.19 0.90 0.30
0.99 0.99 0.99
0.88 0.88 0.88
0.60 0.60 0.27

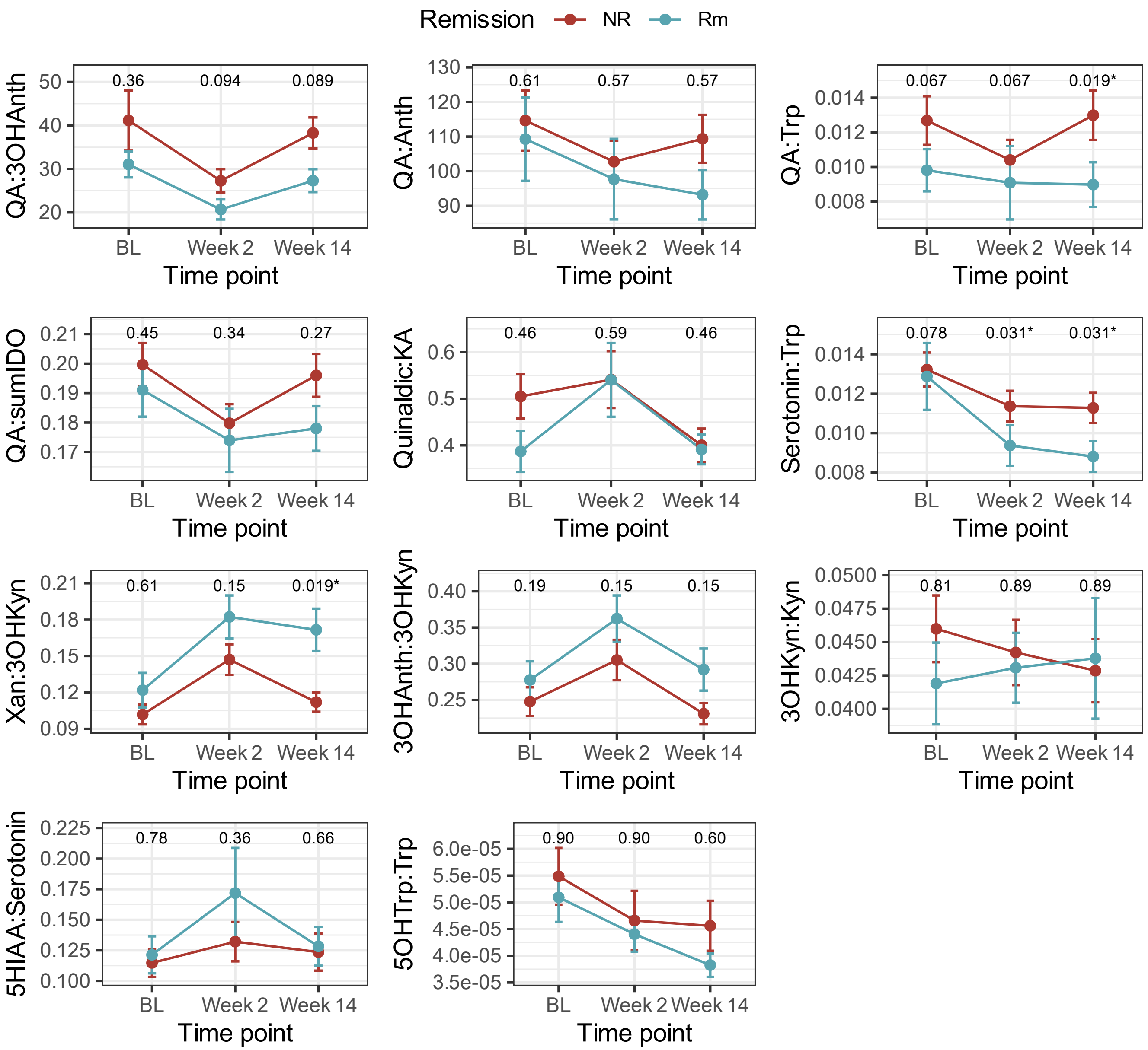

Remission
NR
Rm
QA:3OHAnth
0.36
0.094
0.089
50
40
30
20
QA:Anth
0.61
0.57
0.57
130
120
110
100
90
QA:Trp
0.067
0.067
0.019*
0.014
0.012
0.010
0.008
QA:sumIDO
0.45
0.34
0.27
0.21
0.20
0.19
0.18
0.17
Quinaldic:KA
0.46
0.59
0.46
0.6
0.5
0.4
Serotonin:Trp
0.078
0.031*
0.031*
0.014
0.012
0.010
0.008
Xan:3OHKyn
0.61
0.15
0.019*
0.21
0.18
0.15
0.12
0.09
3OHAnth:3OHKyn
0.19
0.15
0.15
0.40
0.35
0.30
0.25
3OHKyn:Kyn
0.81
0.89
0.89
0.0500
0.0475
0.0450
0.0425
0.0400
5HIAA:Serotonin
0.78
0.36
0.66
0.225
0.200
0.175
0.150
0.125
0.100
5OHTrp:Trp
0.90
0.90
0.60
6.0e-05
5.5e-05
5.0e-05
4.5e-05
4.0e-05
3.5e-05
BL
Week 2
Week 14
Time point

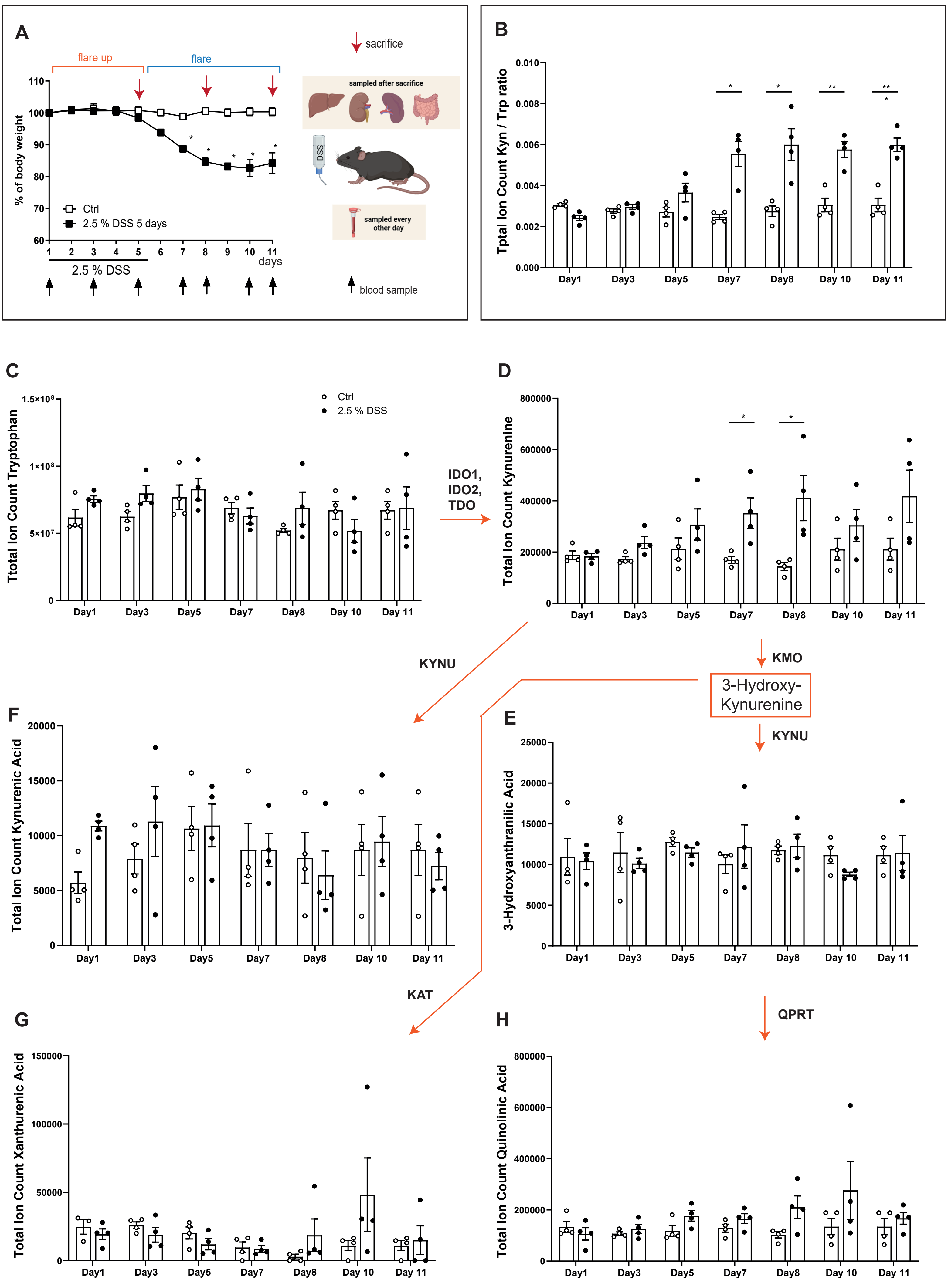

A
flare up
flare
sacrifice
sampled after sacrifice
DSS
sampled every other day
% of body weight
Ctrl
2.5 % DSS 5 days
days
2.5 % DSS
blood sample
B
Tptal Ion Count Kyn / Trp ratio
C
Ttotal Ion Count Tryptophan
Ctrl
2.5 % DSS
IDO1, IDO2, TDO
D
Total Ion Count Kynurenine
KMO
3-Hydroxy-Kynurenine
KYNU
E
3-Hydroxyanthranilic Acid
KYNU
F
Total Ion Count Kynurenic Acid
KAT
G
Total Ion Count Xanthurenic Acid
QPRT
H
Total Ion Count Quinolinic Acid
Day1
Day3
Day5
Day7
Day8
Day 10
Day 11

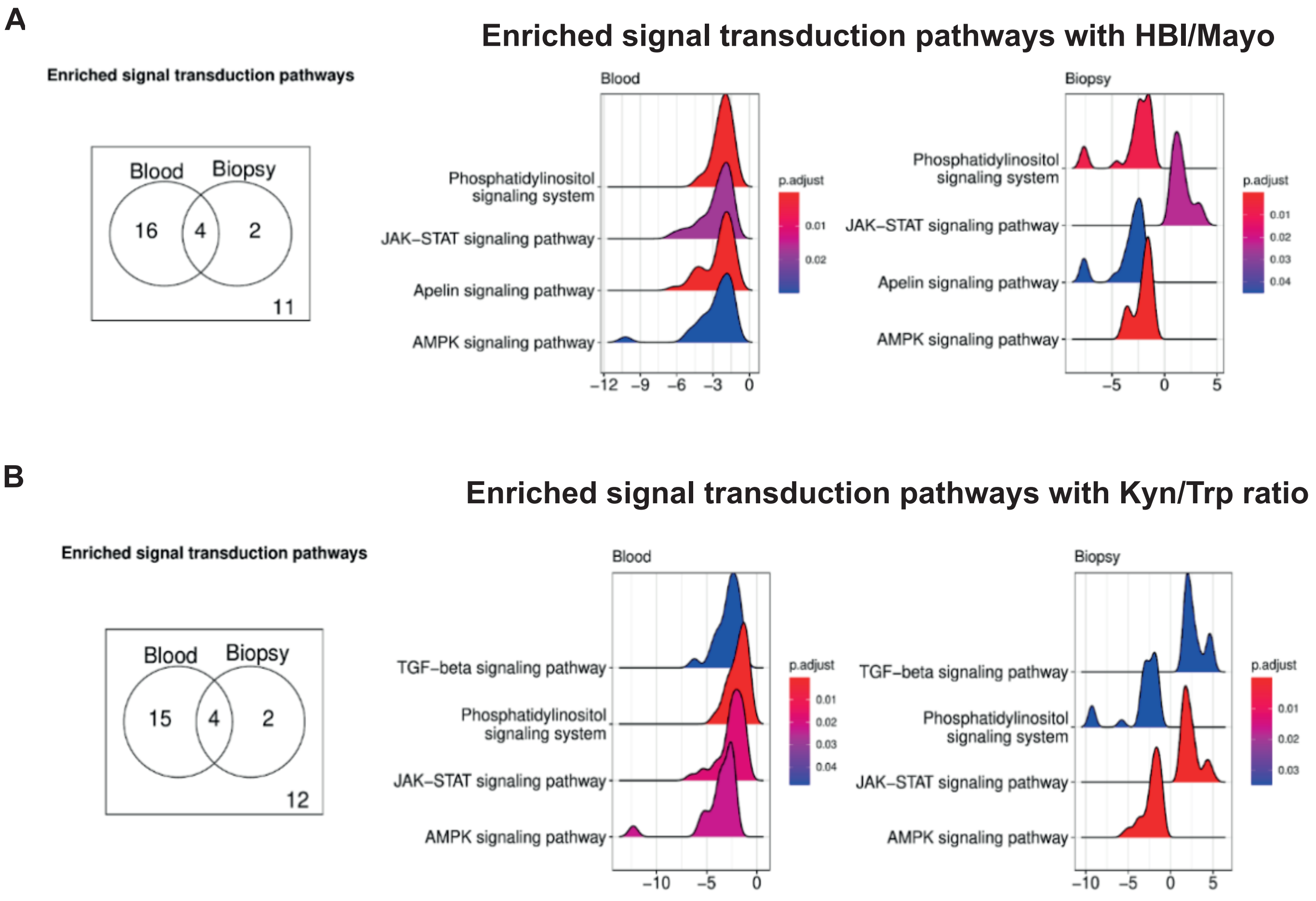
A
Enriched signal transduction pathways with HBI/Mayo
Enriched signal transduction pathways
Blood
Biopsy
16
4
2
11
Blood
Phosphatidylinositol signaling system
JAK−STAT signaling pathway
Apelin signaling pathway
AMPK signaling pathway
−12
−9
−6
−3
0
p.adjust
0.01
0.02
Biopsy
Phosphatidylinositol signaling system
JAK−STAT signaling pathway
Apelin signaling pathway
AMPK signaling pathway
−5
0
5
p.adjust
0.01
0.02
0.03
0.04
B
Enriched signal transduction pathways with Kyn/Trp ratio
Enriched signal transduction pathways
Blood
Biopsy
15
4
2
12
Blood
TGF−beta signaling pathway
Phosphatidylinositol signaling system
JAK−STAT signaling pathway
AMPK signaling pathway
−10
−5
0
p.adjust
0.01
0.02
0.03
0.04
Biopsy
TGF−beta signaling pathway
Phosphatidylinositol signaling system
JAK−STAT signaling pathway
AMPK signaling pathway
−10
−5
0
5
p.adjust
0.01
0.02
0.03

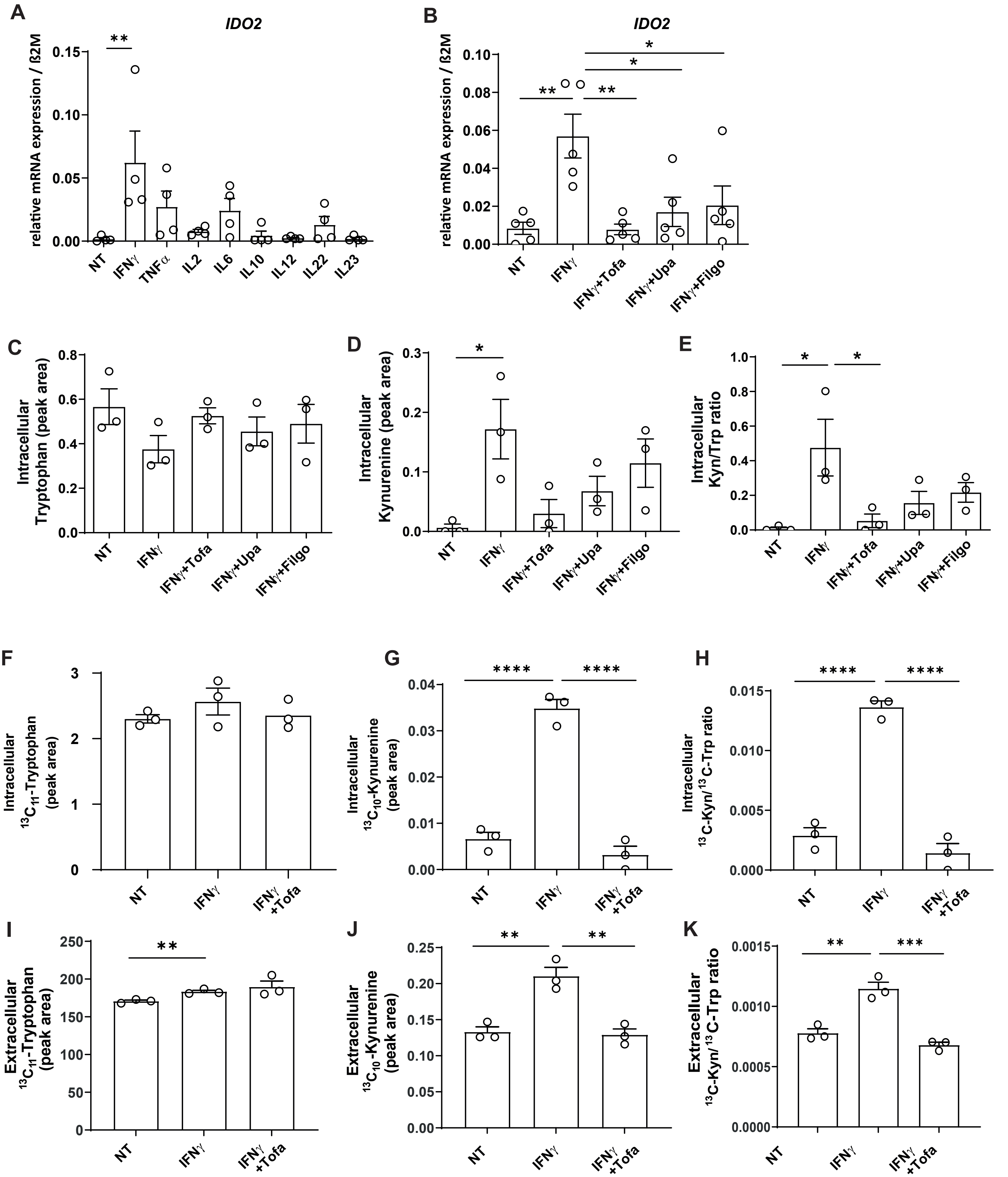

A
IDO2
relative mRNA expression / ß2M
NT
IFNγ
TNFα
IL2
IL6
IL10
IL12
IL22
IL23
B
IDO2
IFNγ+Tofa
IFNγ+Upa
IFNγ+Filgo
C
Intracellular Tryptophan (peak area)
D
Intracellular Kynurenine (peak area)
E
Intracellular Kyn/Trp ratio
F
Intracellular $^{13}C_{11}$-Tryptophan (peak area)
G
Intracellular $^{13}C_{10}$-Kynurenine (peak area)
H
Intracellular ^{13}C-Kyn/^{13}C-Trp ratio
I
Extracellular $^{13}C_{11}$-Tryptophan (peak area)
J
Extracellular $^{13}C_{10}$-Kynurenine (peak area)
K
Extracellular ^{13}C-Kyn/^{13}C-Trp ratio

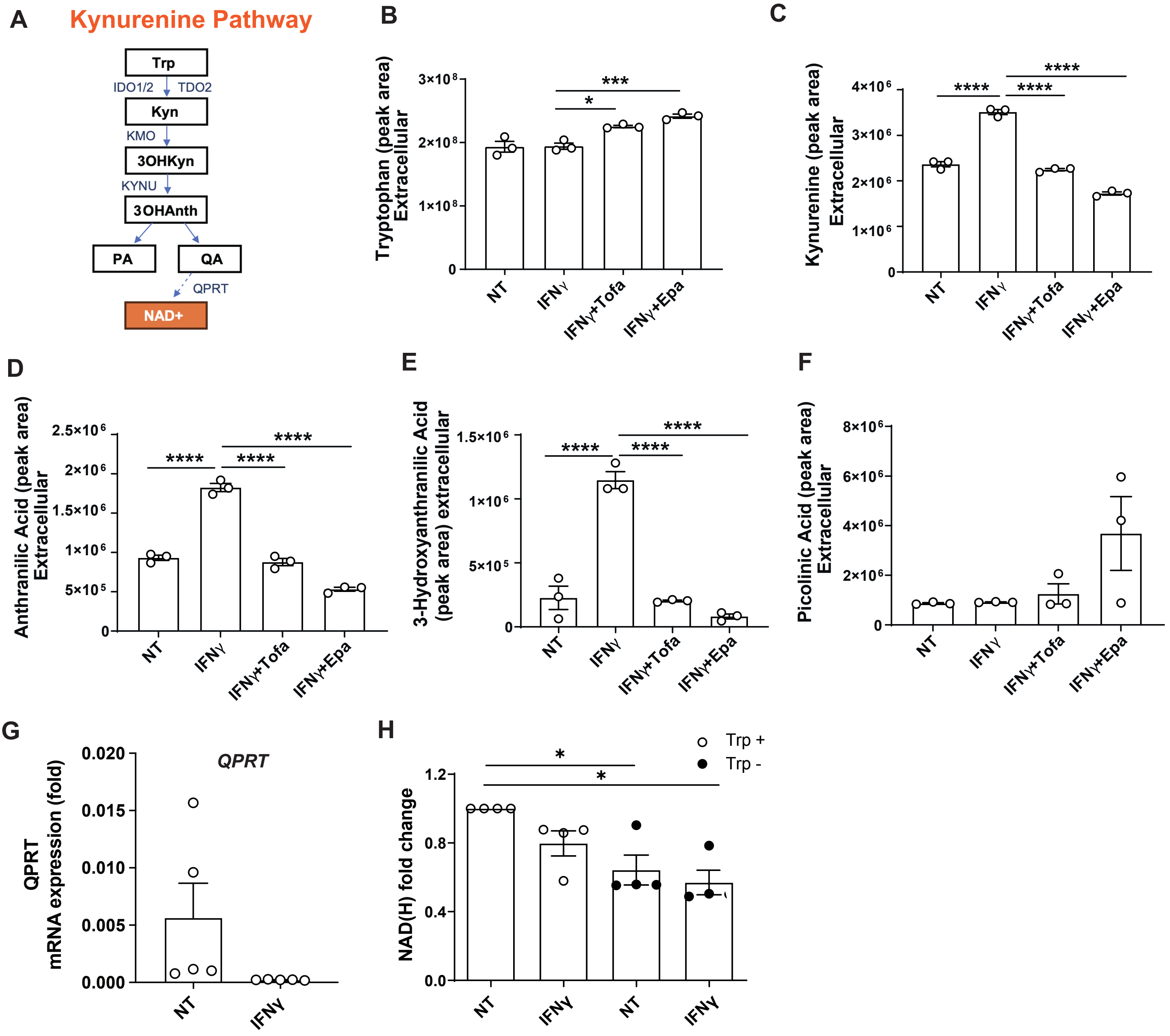
A
Kynurenine Pathway
Trp
IDO1/2
TDO2
Kyn
KMO
3OHKyn
KYNU
3OHAnth
PA
QA
QPRT
NAD+
B
Tryptophan (peak area) Extracellular
NT
IFNγ
IFNγ+Tofa
IFNγ+Epa
C
Kynurenine (peak area) Extracellular
D
Anthranilic Acid (peak area) Extracellular
E
3-Hydroxyanthranilic Acid (peak area) extracellular
F
Picolinic Acid (peak area) Extracellular
G
QPRT
QPRT mRNA expression (fold)
H
Trp +
Trp -
NAD(H) fold change

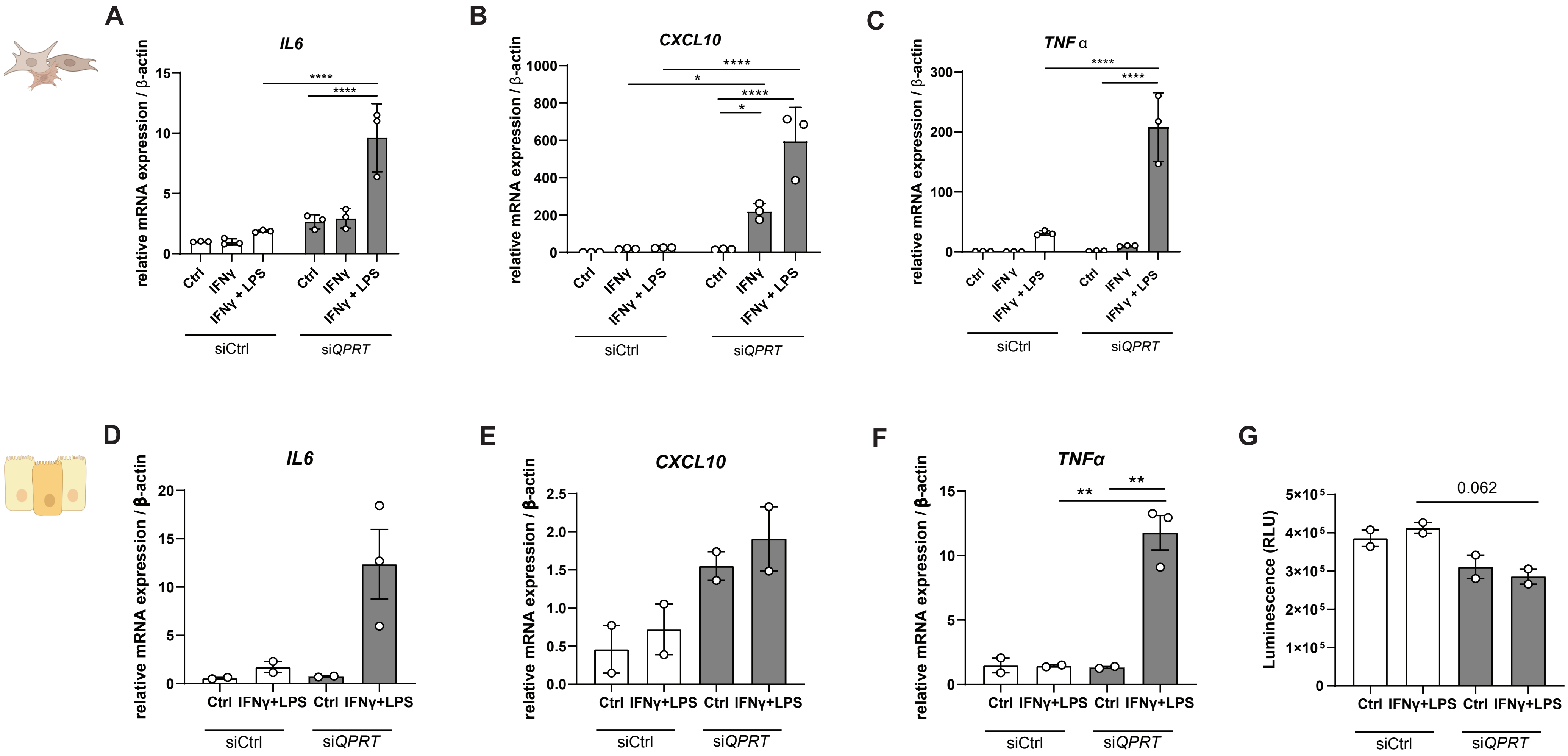
A
IL6
relative mRNA expression / β-actin
0
5
10
15
****
****
Ctrl
IFNγ
IFNγ + LPS
Ctrl
IFNγ
IFNγ + LPS
siCtrl
siQPRT
B
CXCL10
relative mRNA expression / β-actin
0
200
400
600
800
1000
****
*
****
*
Ctrl
IFNγ
IFNγ + LPS
Ctrl
IFNγ
IFNγ + LPS
siCtrl
siQPRT
C
TNF α
relative mRNA expression / β-actin
0
100
200
300
****
****
Ctrl
IFN γ
IFN γ + LPS
Ctrl
IFN γ
IFN γ + LPS
siCtrl
siQPRT
D
IL6
relative mRNA expression / β-actin
0
5
10
15
20
Ctrl
IFNγ+LPS
Ctrl
IFNγ+LPS
siCtrl
siQPRT
E
CXCL10
relative mRNA expression / β-actin
0.0
0.5
1.0
1.5
2.0
2.5
Ctrl
IFNγ+LPS
Ctrl
IFNγ+LPS
siCtrl
siQPRT
F
TNFα
relative mRNA expression / β-actin
0
5
10
15
**
**
Ctrl
IFNγ+LPS
Ctrl
IFNγ+LPS
siCtrl
siQPRT
G
Luminescence (RLU)
0
1×10⁵
2×10⁵
3×10⁵
4×10⁵
5×10⁵
0.062
Ctrl
IFNγ+LPS
Ctrl
IFNγ+LPS
siCtrl
siQPRT

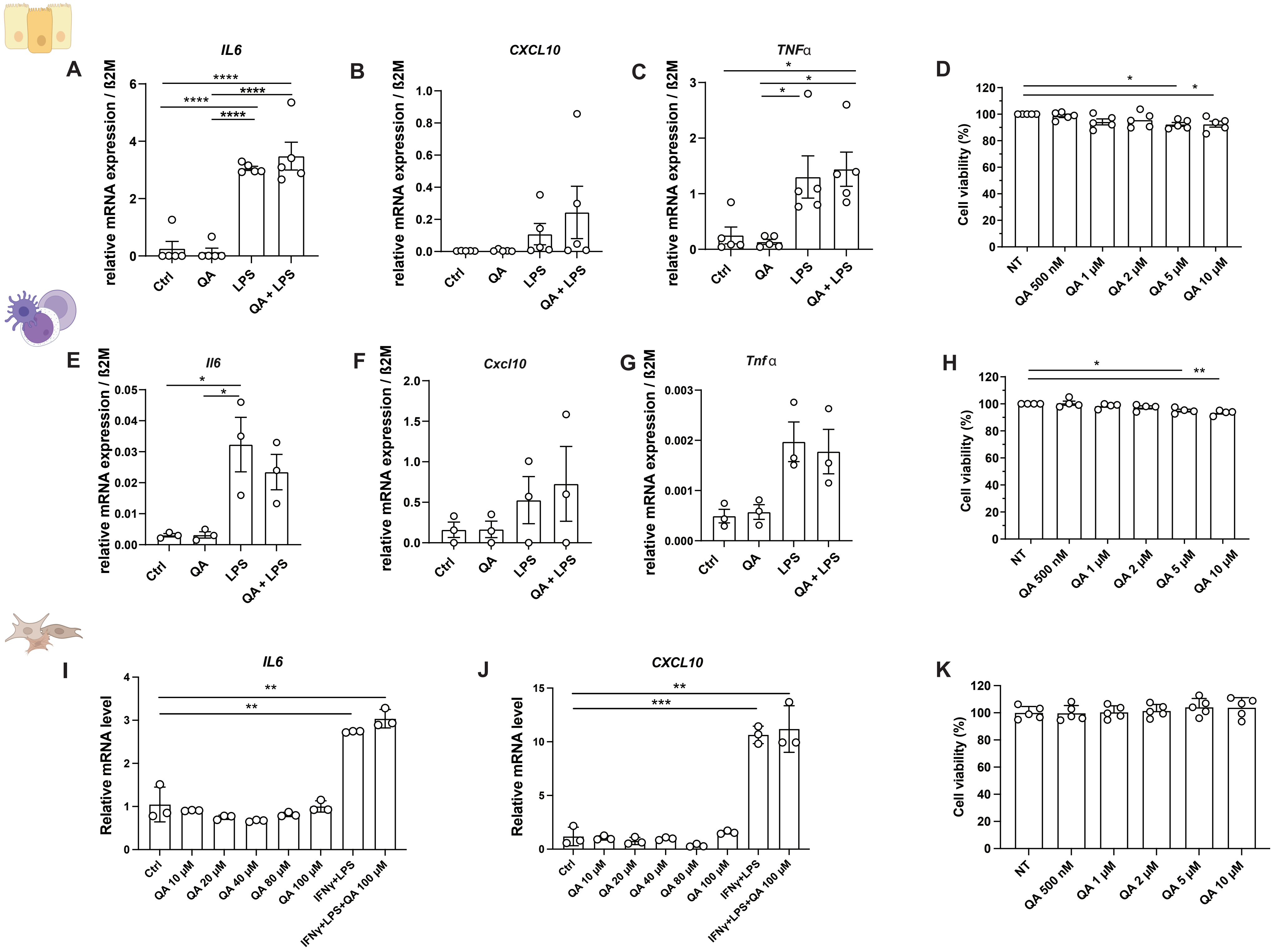

A
IL6
relative mRNA expression / ß2M
Ctrl
QA
LPS
QA + LPS
B
CXCL10
C
TNFα
D
Cell viability (%)
NT
QA 500 nM
QA 1 μM
QA 2 μM
QA 5 μM
QA 10 μM
E
Il6
F
Cxcl10
G
Tnfα
H
I
IL6
Relative mRNA level
QA 10 μM
QA 20 μM
QA 40 μM
QA 80 μM
QA 100 μM
IFNγ+LPS
IFNγ+LPS+QA 100 μM
J
CXCL10
K
